# TDP-43 pathology is associated with increased tau burdens and seeding

**DOI:** 10.1186/s13024-023-00653-0

**Published:** 2023-09-30

**Authors:** Sandra O. Tomé, Grigoria Tsaka, Alicja Ronisz, Simona Ospitalieri, Klara Gawor, Luis Aragão Gomes, Markus Otto, Christine A. F. von Arnim, Philip Van Damme, Ludo Van Den Bosch, Estifanos Ghebremedhin, Celeste Laureyssen, Kristel Sleegers, Rik Vandenberghe, Frederic Rousseau, Joost Schymkowitz, Dietmar Rudolf Thal

**Affiliations:** 1 Laboratory of Neuropathology – Department of Imaging and Pathology, KU Leuven, Leuven, Belgium; 2 Leuven Brain Institute, KU Leuven, Leuven, Belgium; 3 Switch Laboratory, VIB-KU Leuven Center for Brain & Disease Research, Leuven, Belgium; 4 Department of Cellular and Molecular Medicine, KU Leuven, Leuven, Belgium; 5 Department of Neurology, University of Ulm, Ulm, Germany; 6 Department of Neurology, University of Halle, Halle, Germany; 7 Department of Geriatrics, University Medical Center Göttingen, Göttingen, Germany; 8 Laboratory for Neurobiology – VIB-KU Leuven, Leuven, Belgium; 9 Department of Neurology, UZ Leuven, Leuven, Belgium; 10 Institute for Clinical Neuroanatomy – Johann Wolfgang Goethe University, Frankfurt Am Main, Germany; 11 Complex Genetics of Alzheimer’s Disease Group, VIB-University of Antwerp Center for Molecular Neurology, Antwerp, Belgium; 12 Department of Biomedical Sciences, University of Antwerp, Antwerp, Belgium; 13 Laboratory of Experimental Neurology - Department of Neurosciences, KU Leuven, Leuven, Belgium; 14 Department of Pathology, UZ Leuven, Leuven, Belgium

**Keywords:** Alzheimer’s Disease, TDP-43, LATE-NC, Co-pathologies seeding, Tau, Protein aggregation

## Abstract

**Background:**

Most Alzheimer’s Disease (AD) cases also exhibit limbic predominant age-related TDP-43 encephalopathy neuropathological changes (LATE-NC), besides amyloid-β plaques and neurofibrillary tangles (NFTs) containing hyperphosphorylated tau (p-tau). LATE-NC is characterized by cytoplasmic aggregates positive for pathological TDP-43 and is associated with more severe clinical outcomes in AD, compared to AD cases lacking TDP-43 pathology TDP-43: AD(LATE-NC-). Accumulating evidence suggests that TDP-43 and p-tau interact and exhibit pathological synergy during AD pathogenesis. However, it is not yet fully understood how the presence of TDP-43 affects p-tau aggregation in symptomatic AD.

**Methods:**

In this study, we investigated the impact of TDP-43 proteinopathy on p-tau pathology with different approaches: histologically, in a human post-mortem cohort (*n* = 98), as well as functionally using a tau biosensor cell line and TDP-43^A315T^ transgenic mice.

**Results:**

We found that AD cases with comorbid LATE-NC, AD(LATE-NC+), have increased burdens of pretangles and/or NFTs as well as increased brain levels of p-tau199, compared to AD(LATE-NC-) cases and controls. The burden of TDP-43 pathology was also correlated with the Braak NFT stages. A tau biosensor cell line treated with sarkosyl-insoluble, brain-derived homogenates from AD(LATE-NC+) cases displayed exacerbated p-tau seeding, compared to control and AD(LATE-NC-)-treated cells. Consistently, TDP-43^A315T^ mice injected with AD(LATE-NC+)-derived extracts also exhibited a more severe hippocampal seeding, compared to the remaining experimental groups, albeit no TDP-43 aggregation was observed.

**Conclusions:**

Our findings extend the current knowledge by supporting a functional synergy between TDP-43 and p-tau. We further demonstrate that TDP-43 pathology worsens p-tau aggregation in an indirect manner and increases its seeding potential, probably by increasing p-tau levels. This may ultimately contribute to tau-driven neurotoxicity and cell death. Because most AD cases present with comorbid LATE-NC, this study has an impact on the understanding of TDP-43 and tau pathogenesis in AD and LATE, which account for the majority of dementia cases worldwide. Moreover, it highlights the need for the development of a biomarker that detects TDP-43 during life, in order to properly stratify AD and LATE patients.

**Supplementary Information:**

The online version contains supplementary material available at 10.1186/s13024-023-00653-0.

## Background

Transactive response DNA-binding protein 43 kDa (TDP-43) constitutes the major pathological hallmark of amyotrophic lateral sclerosis (ALS) and frontotemporal lobar degeneration with TDP-43 inclusions (FTLD-TDP) [1]. TDP-43 was also found to accumulate in limbic areas of the majority of Alzheimer’s Disease (AD) patients [2–4], in addition to amyloid-β (Aβ) and tau proteins, and has been recently defined as limbic-predominant age-related TDP-43 encephalopathy neuropathological changes (LATE-NC) [5]. Importantly, this co-pathology is associated with smaller hippocampi, worse cognitive decline and a more severe disease outcome in AD patients [6, 7].

Accumulating evidence suggests that TDP-43 and p-tau exhibit pathological synergy during AD pathogenesis, pointing to a shared pathological cascade [8, 9]. Indeed, we and others have observed that symptomatic AD cases with LATE-NC, i.e., AD(LATE-NC+), display higher Braak neurofibrillary tangle (NFT) stages and an increased p-tau burden [6, 10, 11], probably due to common upstream factors that influence both pathologies, such as the APOE ε4 allele [9, 12–14]. Importantly, TDP-43 and p-tau can co-localize in the same neurons and physically interact in AD(LATE-NC+), regardless of dementia status [15–17]. Recent studies using animal models have also observed that TDP-43 is able to modulate tau aggregation, specifically by exacerbating it, leading to increased tau pathology and neurotoxicity [17, 18], however, it is not yet fully clear how TDP-43 impacts p-tau aggregation.

Due to the presence of a “prion-like” glycine-rich domain in the C-terminal region, TDP-43 is prone to self-template and spread to other brain regions [19, 20]. This spread has been well documented in human ALS [21, 22], FTLD-TDP [23, 24] and AD [5, 25]. The injection of patient-derived material in overexpression models has shown to induce pathological seeding, constituting a reliable model to study protein aggregation in vivo. Studies have shown that human AD-derived tau [26, 27] as well as FTLD-TDP-derived TDP-43 seeds [28] are able to seed in the mouse brain, recapitulating the proteopathic spreading patterns in the brains of AD and FTLD-TDP brains, respectively. Considerable efforts have been made to study TDP-43 aggregation and seeding properties in vitro [20, 29]. However, it is not yet clear which mechanisms drive AD-derived TDP-43 and tau pathogenesis*.*

Here, we investigated the impact of TDP-43 pathology on tau aggregation in symptomatic AD by investigating a cohort of 98 human autopsy cases as well as using in vivo and in vitro approaches.

We report that AD(LATE-NC+) cases exhibit increased burdens of p-tau as well as increased brain levels of p-tau199. We also observed that cells and animals exposed to AD(LATE-NC+) brain lysates exhibited increased p-tau seeding compared to AD cases lacking LATE-NC and control extracts. Overall, our findings demonstrate that TDP-43 worsens p-tau pathology, which is then reflected in higher burdens and increased seeding potential of p-tau in the brains of AD(LATE-NC+) patients.

## Methods

### Human autopsy cases

A total of 98 human autopsy cases were used in this study, including 26 non-demented controls without AD neuropathological changes (ADNC), 67 demented AD cases, 55 of which exhibiting comorbid LATE-NC and 5 FTLD-TDP (subtypes A, B or C [30]) cases (Table 1). Among these, brains from 34 cases were used for biochemical analyses and in vitro experiments and brain tissue from 4 cases was used for stereotaxic injections in vivo (Table 2).

**Table 1:** Characteristics of the human post-mortem cases used in the study (*n* = 98: 26 controls, 12 AD (LATE-NC-), 55 AD(LATE-NC+), 5 FTLD-TDP). NA = non-applicable or not available. Results are displayed in mean ± standard deviation except gender and APOE frequencies

**Variable**	**Group**			
	Controls	AD(LATE-NC-)	AD(LATE-NC+)	FTLD-TDP
Age	64.77 ± 9.15	80.42 ± 9.68	78.75 ± 9.97	61.67 ± 13.67
Gender frequency (m/f)	84.6%/15.4%	50%/50%	43.6%/56.4%	60%/40%
Braak NFT Stage	0.54 ± 0.65	3.50 ± 1.09	5.16 ± 1.08	0.83 ± 0.41
Aβ Phase	0	4.60 ± 0.52	4.72 ± 0.69	0
LATE-NC Stage	0.5 ± 0.82	0	2.22 ± 0.54	NA
Braak LBD Stage	0.35 ± 1.23	0.25 ± 0.86	1.43 ± 2.33	0
CERAD Score	0	1.17 ± 0.72	2.42 ± 0.74	0
CDR score	0.17 ± 0.48	1.73 ± 0.90	2.59 ± 0.69	2.50 ± 0.55
NIA-AA score	0	1.92 ± 0.51	2.69 ± 0.58	0
APOE ε4 frequency	11.5%	20%	56.55%	16.7%
APOE ε2 frequency	12%	0%	8.7%	NA

**Table 2:** Characterization of human cases used for stereotactical injection. NA = non-applicable

Neuropathological Diagnosis	Injection group	Age	Sex	Aβ phase	Braak NFT stage	LATE-NC stage	CERAD core	NIA-AA score	CDR score
PART	Control	76	m	0	I	0	0	1	1
ADNC	AD(LATE-NC-)	54	f	5	VI	0	2	3	3
ADNC, LATE-NC	AD(LATE-NC+)	74	m	5	VI	2	2	3	2
FTLD-TDP type C	FTLD-TDP	62	f	0	0	NA	0	0	3

The brains were collected at university and municipal hospitals in Ulm (Germany) and Leuven (Belgium) as well as well as from the brainbank donated for research by GE Healthcare after the phase III flutemetamol autopsy study had concluded [31]. Autopsies were performed in accordance with German/Belgian law after approval by the ethical committees from Ulm (Germany, study number 54/08) and UZ Leuven (Belgium, study numbers S-59292, S-52791, S-66705). The samples received from GE-Healthcare clinical trials were recruited after ethical approval (ClinicalTrials.gov identifiers NCT01165554, and NCT02090855).

Phases of Aβ plaque deposition according to Thal et al. were assessed as described previously, based on anti-Aβ_17-24_ stained brain sections [32]. Braak stages for NFTs spread in the brain were determined as previously described based on anti-p-tau202/205 immunostained sections [33]. The neuropathological diagnosis of ADNC was performed as published by the National Institute of Aging and Alzheimer Association working group (NIA-AA criteria) [34]. LATE-NC was diagnosed and staged according to the recently published guidelines from a consensus working group [5, 35]. Briefly, LATE-NC was considered if neuronal cytoplasmic inclusions, tangle-like inclusions or dystrophic neurites positive for pTDP-43 (409/410, 1:5000, #22309–1-AP, Proteintech, Rosemont, IL) were observed in one or more of the following regions: amygdala, posterior hippocampus and frontal cortex. Global Clinical dementia rating (CDR) scores were retrospectively assessed according to previously established guidelines [36].

Stages of α-synuclein pathology (= Braak LBD stages) were determined according to Braak et al., 2003 [37]. Briefly, in stages 1 and 2, the pathology is mostly confined to the medulla oblongata. The midbrain becomes affected in stage 3 and the temporal mesocortex and allocortex are affected at stage 4. Stages 5 and 6 are characterized by α-synuclein pathology in neocortical areas.

APOE ε4 genotypes of these cases were obtained as described previously [38]. DNA was extracted from fresh frozen or formaldehyde-fixed, paraffin-embedded tissue and PCR was performed followed by enzymatic digestion.

### TDP-43^A315T^ mouse model

Heterozygous mice overexpressing a TDP-43 construct with the C-terminal A315T mutation driven by the mouse prion protein (PrP) promoter were used in this study, originally developed by Wegorzewska et al. [39]. Transgenic mice were bred by continuous backcrossing of heterozygous males with wild-type females on a C57BL/6 background. Sixty-six transgenic mice distributed over five groups (*n* = 5–10 per group) were injected with human brain-derived homogenates from different cases (Table 3).

**Table 3:** Experimental mouse groups with respective protein concentrations which have been stereotactically injected. Injection volume = 2,5µL

Group	Total protein (µg/mL)	Total protein per site (µg)	p-tau181 (pg/mL)	p-tau199 (pg/mL)	p-tau181 per site (pg)	p-tau199 per site (pg)	Total TDP-43 (ng/mL)	TDP-43 per site (pg)
Control	582	1,46	186,54	13,10	0,47	0,03	113,82	284,55
AD (LATE-NC-)	700	1,75	34198,23	6946,48	85,50	17,37	1482,12	3705,30
AD (LATE-NC +)	826	2,07	177256,20	17700,70	443,14	44,25	1157,58	2893,95
FTLD-TDP	1220	3,05	4022,57	180,91	10,06	0,45	3637,94	9094,85

After 2 months of age, the animals were given gel food (DietGel®31M, ClearH2O, Portland, ME, US) until they were sacrificed. This overcame known intestinal obstruction problems and metabolic problems associated with this model [39, 40], and significantly extended their lifetime, up to 16 months [40, 41]. Only 5 out of 59 animals used for experiments displayed motor symptoms upon end of the experiment. Thus, the gross majority of the animals were not diseased at time of death.

All animal care and experiments were approved by the KU Leuven Ethical Committee (P126/2018) and carried out according to the Belgian law.

### Protein extraction and BCA assay

For in vitro assays, sarkosyl-insoluble homogenates from the entorhinal and frontal cortices were used for all groups. For in vivo injections, the entorhinal cortex region was selected for the control, AD(LATE-NC+) and FTLD-TDP group, as it is a region with common co-occurrence of TDP-43 and p-tau pathologies [42]. The occipital cortex region from a Braak NFT stage VI was selected for the AD(LATE-NC-) group, in order to control for the presence of p-tau and absence of TDP-43 pathologies, respectively.

Proteins were extracted using a previously established protocol [28], undergoing sequential centrifugations with buffers of increasing strengths. Briefly, grey matter was solubilized in a high-salt buffer (10mM Tris–HCL, 0.5M NaCl, 10% sucrose, 1mM DTT, 1% Triton-X and a cocktail of protease/phosphatase inhibitors (Halt, ThermoFisher Scientific) followed by ultra-centrifugation. Myelin was removed in 20% sucrose in a high-salt buffer, after ultra-centrifugation. The pellet was further resuspended in 2% sarkosyl in high-salt buffer, containing Pierce Universal Nuclease (0.1ug/mL, Thermofisher Scientific) for 45 min and ultra-centrifuged. The resulting pellet was washed twice in PBS. The final pellet (sarkosyl-insoluble fraction) was resuspended in 300µL of PBS, sonicated and used for ‘sandwich’ ELISA assays, in vitro assays and as injection fractions. The extraction protocol is depicted in Suppl. Figure 5a, Additional file 1. The total protein was quantified using the Pierce® BCA Protein assay Kit (ThermoFisher Scientific), according to the manufacturer’s instructions.

### Stereotactic injections

The mice received injections of brain lysates at 12 months of age. The animals were anaesthetized by intraperitoneal injection of ketamine 75 mg/kg and medetomidine 1mg/kg. After placing the mice in a stereotactic frame (72–6049, Harvard Apparatus, Massachusetts, USA), 2,5 μl of brain lysates were injected in the left hippocampal formation (-2,5 mm AP, + 2 mm LR, -1,8 mm DV) using a Hamilton syringe (Ref: HH 7635–01). The injection was performed at a speed of 1 μl/minute and the needle was left for an additional five minutes after injection. Anesthesia was reversed with Atipamezole. The mice received a subcutaneous injection of Buprenorphine for pain reduction after surgery. After 4 months of inoculation period, mice were euthanized at 16 months of age by decapitation under anesthesia with ketamine and medetomidine. The mouse brains and spinal cords were extracted and kept in 4% paraformaldehyde for approximately four days. After paraffin embedment, serial sections of 5 μm were cut with a microtome and analyzed by immunohistochemistry.

### Immunohistochemistry

Human and mouse brain sections were deparaffinized and processed with citrate buffer for epitope retrieval (pH = 6, EnvisionTM Flex Target Retrieval Solution, Dako, K8005) for 10 min. Endogenous mouse peroxidase was blocked for 5 min in order to avoid unspecific reactions in all slides.

Human sections were stained overnight with the rabbit polyclonal anti-pTDP-43 (409/410, dilution 1:5000, Proteintech #22309–1-A, Rosemont, IL, USA), rabbit polyclonal anti-C-terminal TDP-43 (260-414aa., dilution 1:1000, Proteintech #12892–1-AP, Rosemont, IL, USA), mouse monoclonal anti-p-tau202/205 (dilution 1:1000, clone AT8, #MN1020, ThermoFisher Scientific, Waltham, MA, USA) and mouse monoclonal anti p-tau181 (dilution 1:5000, clone AT270, #MN1050, ThermoFisher Scientific). A secondary anti-rabbit or anti-mouse HRP antibody was applied for 30 min (Vector Laboratories, Newark, NJ, USA).

The mouse sections underwent a mouse-on-mouse immunohistochemistry protocol allowing the staining of mouse tissue with anti-mouse antibodies was performed based on a previously described protocol [43]. Then, a protein block using normal goat serum was applied for 10 min. To avoid unspecific reaction with endogenous mouse IgGs, mouse monoclonal p-tau202/205 primary antibody was coupled with a biotinylated Fab fragment and incubated overnight. Next, streptavidin-HRP (1:200) was applied to the slides for 30 min in order to detect the biotinylated Fab fragment.

3,3’-diaminobenzidine (DAB) was used as a chromogen to yield brown reaction products in all sections. Counterstaining was performed with hematoxylin.

### Histological quantifications

For *human* cases, three representative 200 × fields in the CA1 subregion of the hippocampus and the frontal cortex (layers III-IV) were imaged and quantified, respectively. For this, the percentage of affected neurons was calculated by performing a ratio of the average number of positive neurons over the total average number of neurons in the three fields. For p-tau202/205, NFTs and pretangles were considered, whereas for TDP-43, pTDP-43-positive neuronal cytoplasmic inclusions (NCIs) and neurofibrillary tangle-like material were considered, as done previously [10]. These quantifications were performed manually with ImageJ® (multipoints).

For *mouse* tissue samples, two different quantifications were performed. First, the spatial expansion of p-tau lesions from anterior to posterior hippocampus was determined. For this, 12 coronal sections covering the entire hippocampal region were stained with p-tau202/205 and scored as negative or positive, based on the absence or presence of p-tau pathology, respectively. This was considered as hippocampal anterior posterior score (hAP score) and was calculated as a percentage of the sum of affected sections and represents the propagation speed of p-tau seeds, as done previously [44]. This score directly reflects the spread of the p-tau seeds in the mouse hippocampus. Second, to analyze the local p-tau severity, a hotspot section from each animal where the seeding was most prominent was analyzed. Here, the total number of particles was measured (ImageJ®, threshold setting) in an image of 50 × magnification. The percentage of C-t TDP-43 nuclear clearance was performed with manual counts using ImageJ® (multipoints) with one microscopic field of 200 × magnification for CA1-hippocampus and motor cortex in a Leica (Wetzlar, Germany) microscope.

Neuronal density in *human and mouse* tissue samples was quantified using 200 × magnification fields, using stained sections with anti-pTDP-43 or C-t TDP-43, respectively. Neurons were identified based on morphology and the presence of a clear nucleolus. The total number of neurons was quantified in each field and results were normalized to neurons per mm^2^.

### Immunoprecipitations

100ug of total protein of sarkosyl-insoluble extracts were used per reaction. The immunoprecipitation protocol was performed as previously described [15]. Briefly, 50µL of rabbit magnetic Dynabeads® (Thermofisher Scientific) were washed three times and incubated overnight at 4°C with 2ug of the primary antibody (pTDP-43 409/410, #22309–1-AP, Proteintech, Rosemont, IL) per reaction. Negative IP controls were performed, with 50µL of magnetic beads and 100µg of protein of one non-disease control and one AD case. Next, the beads were washed three times and incubated with each sarkosyl-insoluble sample for 2h at room temperature. The unbound fraction was kept as the “pTDP-43 depleted fraction” whereas the eluent containing the pTDP-43-bound epitopes was denaturated in 2 × LDS sample buffer and kept as the “bound” fraction. The total protein of the depleted fractions was calculated through BCA assay. Both fractions as well as the input were characterized through western blot.

### Western blot

10µg of total protein of sarkosyl-insoluble homogenates or the pTDP-43 depleted fractions resulting from the immunoprecipitation were loaded into NuPAGE 4–12% gels, after protein denaturation with LDS and a reducing agent. The electrophoresis was performed using MOPS-SDS running buffer and proteins were transferred into 0.2 µm nitrocellulose membranes (GE Lifesciences). The membranes were then blocked and incubated overnight at 4°C with rabbit polyclonal anti-TDP43 (350-414aa., Thermofisher Scientific) or mouse monoclonal anti-p-tau 396/404 (gift from Peter Davies). Membranes were washed and incubated with the appropriate secondary antibodies for 1 h at room temperature. Immunoblots were washed and detected using the SuperSignal™ WestDura substrate (ThermoFisher Scientific) and an Amersham Imager 600 (GE Life Sciences, Chicago, IL, USA).

### Enzyme-Linked Immunosorbent Assays (ELISAs)

To quantify the concentration of p-tau and TDP-43 in the human brain homogenates, ELISA assays were carried out using commercially available kits (p-tau199 human ELISA Kit, ThermoFisher Scientific, #KHO0631 and human full-length (1-300aa.) TDP-43 ELISA Kit, Proteintech, #KE00005), according to the manufacturer instructions. Duplicates were performed for all samples. The final concentrations were interpolated based on the polynomial range of the standard curve of the respective assays, using GraphPad Prism software 9.3.1 (471). The interpolated concentrations as well as definitive p-tau and TDP-43 amounts injected per site are displayed in Table 3.

### Tau biosensor cell line

The tau repeat domain (RD) P301S FRET Biosensor (HEK-293) cell line, bought from ATCC (CRL-3275), stably expresses tau RD P301S-CFP and tau RD P301S-YFP. Transfection with tau seeds nucleates the aggregation of the endogenous tau reporter proteins, producing a FRET signal [26]. For seeding experiments, the cells were cultured in DMEM medium, supplemented with 10% FBS, 1 mM sodium pyruvate and non-essential amino acids (Gibco), under an atmosphere of 5% CO_2_ at 37°C. Cells were plated at 5.000 cells/well in poly-L-Lysine-coated 384-well PhenoPlates (PerkinElmer). After 16h, cells were transfected with brain extracts using lipofectamine 3000 (Thermofisher Scientific) according to the manufacturer’s protocol. Before the transfection, the samples were sonicated for 15 min (30 s on, 30 s off at 10A) with a Bioruptor Pico (Diagenode). Each sample (1:20 dilution in Opti-MEM) was mixed with 3000 reagent and added to a mixture of Opti-MEM medium (Gibco) with Lipofectamine 3000. After a 15 min incubation at room temperature, 4μL of mixture was added per well in a total volume of 40μL. After 48h, cell medium was replaced with 40μL 4% formaldehyde and cells were incubated for 5 min, then washed three times with PBS. Nuclear staining was performed with DAPI (Thermofisher D1306) diluted (1:5000 from a stock of 5 mg/mL) in 1% BSA in PBS for 30 min. Three individual plate preparations were performed per sample as independent experiments (*n* = 3). High-content screening was performed using an Operetta (PerkinElmer) equipped with proper filter channels to track tau aggregation through the FRET signal. Image storage (10 fields in 4 planes at a 40 × magnification were acquired per well) and segmentation analysis was performed using the Columbus Plus digital platform (PerkinElmer).

### Statistical analysis

All statistical analyses were performed using GraphPad Prism 9.3.1 (471) software or IBM SPSS 28. Multiple linear regressions were used for comparisons of the human and in vivo data. Simple linear regressions were used to analyze how pTDP-43 pathology predicts p-tau pathology in the CA1 and frontal cortex in human cases. Kruskal–Wallis test with post-hoc Dunn’s test, one-way ANOVA with post-doc Tuckey’s test or Wilcoxon paired-matches signed rank rest were used to analyze in vitro data.

## Results

### Tau pathology is increased in AD cases with LATE-NC

**Fig. 1:**
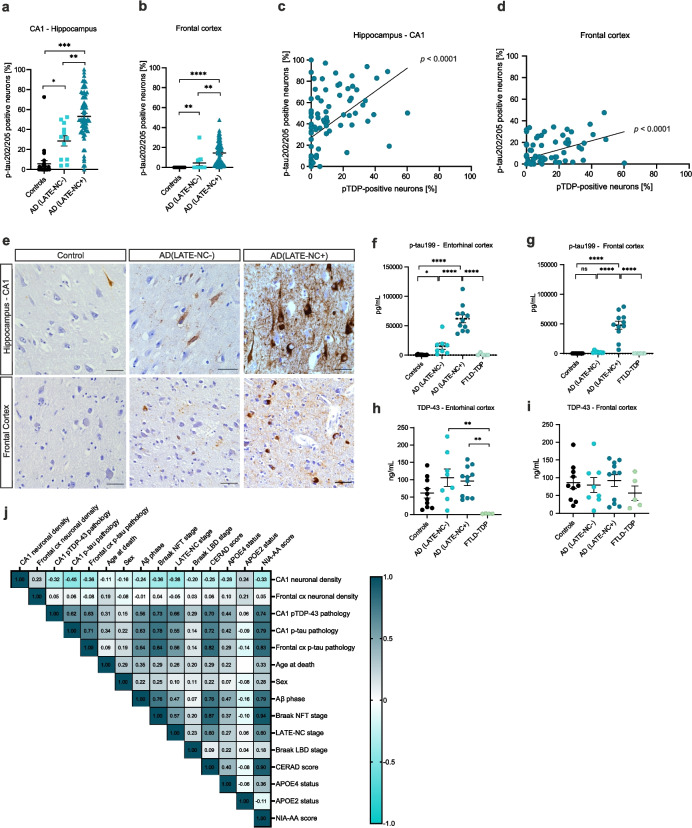
Pathological tau expression is increased in AD cases with comorbid LATE-NC. Immunohistological quantification of the percentage of p-tau202/205 positive neurons (tangles and pretangles) shows that p-tau pathology is significantly increased in AD(LATE-NC+), *n* = 55 compared to controls (*n* = 26) and AD(LATE-NC-) cases (*n* = 12) in the (**a**) CA1 subfield of the posterior hippocampus and compared to controls in (**b**) frontal cortex. Multiple linear regressions controlled for age and sex were used to compare the three neuropathological subgroups. Simple linear regressions in the whole cohort show that hippocampal pTDP-43 pathology is significantly associated with (**c**) hippocampal and (**d**) cortical p-tau pathology. Overview of p-tau202/205 immunostainings in the hippocampus and frontal cortex of a control, AD(LATE-NC-) and AD(LATE-NC+) case is shown in (**e**), 200 × magnification pictures and scale bars = 50µm. p-tau199 levels in sarkosyl-insoluble homogenates of the (**f**) entorhinal cortex or (**g**) frontal cortex of 10 controls, 8 AD(LATE-NC-), 11 AD(LATE-NC+) and 5 FTLD-TDP show that AD(LATE-NC +) cases have increased p-tau199 in both regions compared to controls, AD(LATE-NC-) and FTLD-TDP cases. Physiological TDP-43 levels are significantly decreased in the entorhinal cortex of FTLD-TDP cases, compared to the AD groups (**h**). Levels of total TDP-43 are similar among groups in the frontal cortex (**i**). Multiple linear regressions were used to compare the three neuropathological subgroups. Spearman correlation analyses in the whole cohort including all variables analyzed is depicted in (**j**), coefficient values are displayed; blank cells mean coefficient value < 0.01

To investigate the impact of TDP-43 on tau pathology in AD cases, we investigated 98 post-mortem human cases, including 26 controls without AD, 62 demented cases with moderate to high levels of ADNC (of these, 55 fulfilled the criteria for AD(LATE-NC+) and 12 for AD(LATE-NC-)) and 5 FTLD-TDP cases. First, we performed immunohistological studies to quantify the severity of p-tau in two different brain regions: the CA1 subfield of the posterior hippocampus, as well as the middle-frontal cortex. We quantified the percentage of neurons positive for p-tau202/205-containing tangles and pretangles in controls, AD (LATE-NC-) and AD(LATE-NC+) cases. For validation purposes, we also immunostained 5 controls, 5 AD (LATE-NC-) and 5 AD(LATE-NC+) cases with anti p-tau181 (Suppl. Figure 1, Additional file 1). We then performed multiple linear regression models controlled for age, sex and APOE ε4 status with p-tau pathology (in the CA1 or frontal cortex) as the independent variable. We found that AD(LATE-NC+) cases exhibited significantly increased p-tau pathology in both regions when compared to controls (CA1, p = 0.0003; frontal cortex *p* < 0.0001) and to AD (LATE-NC-) cases (CA1, *p* = 0.0066; frontal cortex, *p* = 0.0097, Fig. 1 a-b, e), even after correcting for age and APOE ε4 status. The number of dystrophic neurites was also exacerbated in the AD(LATE-NC+) group, however this was not quantified (Fig. 1e). When performing simple linear regressions in the whole cohort independently from neuropathological grouping, we observed that the burden of hippocampal pTDP-43 pathology is associated with the burden of hippocampal and cortical p-tau (*p* < 0.0001, *R*^*2*^ = 0.24) (Fig. 1c-d). To validate these data biochemically, we extracted sarkosyl-insoluble homogenates from the entorhinal and frontal cortices of 10 controls, 8 AD(LATE-NC-), 11 AD(LATE-NC+) and 5 FTLD-TDP cases. We then measured the levels of p-tau199 and total TDP-43 (1-300aa.) in the brain extracts through ‘sandwich’ ELISA assays. We observed that p-tau199 protein levels were increased in AD(LATE-NC+) in both brain regions compared to controls (entorhinal and frontal cortex, *p* < 0.0001), AD (LATE-NC-) (entorhinal, *p* = 0.0043; frontal cortex, *p* < 0.0001) and FTLD-TDP cases (entorhinal and frontal cortex, *p* < 0.0001), when performing multiple linear regression models (Fig. 1f-g). The levels of total (physiological) TDP-43 were decreased in FTLD-TDP cases compared to AD(LATE-NC-) and AD(LATE-NC+) in both regions, reaching significance in the entorhinal region (*p* = 0.0028 and *p* = 0.0018, respectively; multiple linear regressions) (Fig. 1h-i). This points to a loss-of-function of nuclear TDP-43 in FTLD-TDP cases.

Of note, the mean Aβ phase and Braak LBD stage were similar among AD groups (Table 1).

Finally, we performed a Spearman correlation analysis covering all controls and AD cases. pTDP-43 and p-tau pathologies were inversely correlated with hippocampal but not cortical neuronal density (*p* = 0.002 and *p* < 0.001, Fig. 1j). Corroborating our previous data, pTDP-43 pathology was significantly associated with both hippocampal and cortical p-tau pathology (*p* < 0.0001). Additionally, pTDP-43 pathology was associated with Braak NFT stages (*p* < 0.0001), Aβ phases (*p* < 0.0001), LBD stage (*p* = 0.007), age at death (*p* < 0.003), APOE ε4 status (*p* < 0.0001), CERAD and NIA-AA scores (*p* < 0.0001, Fig. 1j).

The distribution of LATE-NC stages among our cohort are shown in Suppl. Figure 2, Additional file 1. All models, corresponding *p*-values and correlation coefficients are displayed in Suppl. Tables 1–13, Additional file 1.

### The presence of LATE-NC is associated with exacerbated tau seeding in a tau biosensor cell line

**Fig. 2:**
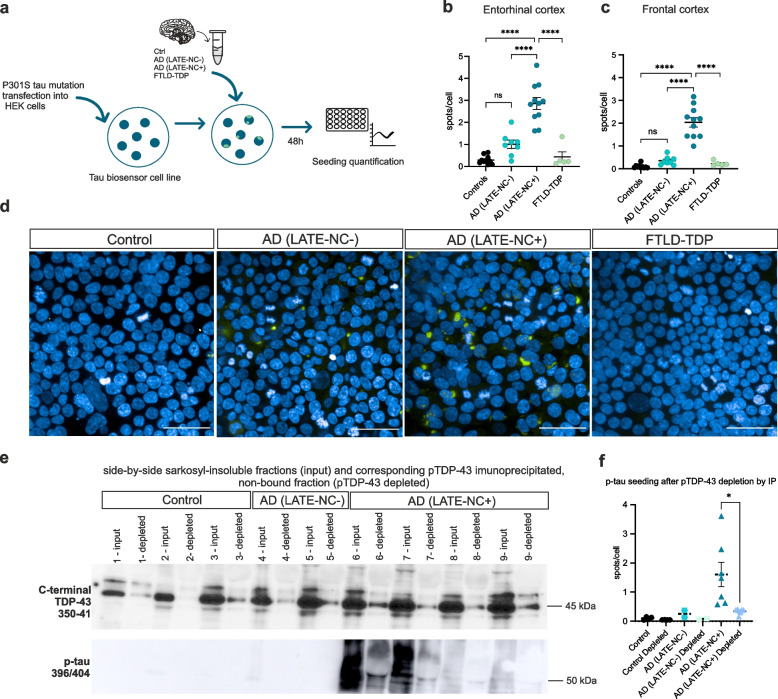
The presence of LATE-NC associates with exacerbated tau seeding in vitro. A tau (P301S) biosensor cell line was transfected with sarkosyl-insoluble homogenates of entorhinal and frontal cortex from 5 controls, 8 AD(LATE-NC-), 11 AD(LATE-NC+) and 5 FTLD-TDP cases. After 48 h, the number of spots/cell (p-tau signal) were automatedly quantified (**a**). At a dilution of 1:20, cells treated with entorhinal cortex extracts from AD(LATE-NC+) cases showed increased p-tau seeding compared to FTLD-TDP and control-treated extracts (**b**). Similarly, cells transfected with frontal cortex homogenates from AD(LATE-NC+) cases display increased seeding compared to controls, AD(LATE-NC-) and FTLD-TDP treated cells (**c**). One-way ANOVA with Tuckey’s correction was used. Overview of each experimental condition is displayed in (**d**), scale bars = 50µm. Immunoprecipitation of pTDP-43 (S409/S410) from sarkosyl-insoluble homogenates shows a significant decrease of TDP-43 (350-414aa., ThermoFisher Scientific) and p-tau396/404 in the non-bound fractions (**e**). When cells are treated with pTDP-43-depleted fractions from 4 controls, 2 AD(LATE-NC-) and 7 AD(LATE-NC+), p-tau seeding was significantly reduced in the AD(LATE-NC+) group, compared to cells treated with the corresponding non-depleted homogenate (**f**), Wilcoxon matched-pairs signed ranked test

In order to functionally address the hypothesis that TDP-43 aggravates p-tau pathogenesis, we performed seeding assays using a previously established tau biosensor cell line [26]. The cell line was transfected with sarkosyl-insoluble homogenates from the entorhinal and frontal cortices of 10 controls, 8 AD(LATE-NC-), 11 AD(LATE-NC+) and 5 FTLD-TDP cases. The seeding efficiency of the different extracts as quantified as the number of fluorescent puncta (spots) per cell, which represent tau aggregates (Fig. 2a). Cells exposed to entorhinal cortex extracts from AD(LATE-NC+), but not AD(LATE-NC-), showed an augmented number of tau aggregates compared to cells treated with control-derived homogenates (*p* < 0.0001, Fig. 2b, d and Suppl. Table 14, Additional file 1). Notably, when cells were transfected with frontal cortex extracts from AD(LATE-NC+), tau seeding was also significantly increased compared to AD (LATE-NC-) (*p* < 0.0001) and to control-treated cells (*p* < 0.0001) (Fig. 2c-d, Suppl. Table 15, Additional file 1). Control and FTLD-TDP homogenates from both brain regions caused only marginal levels of tau seeding (Fig. 2b-c).

Because AD(LATE-NC+) cases show increased levels of p-tau (Fig. 1f-g), we tested whether the exacerbated seeding effects with these homogenates were due to higher p-tau concentrations in these cases. To address this, we performed pTDP-43 immunoprecipitation assays with frontal cortex homogenates of 3 controls, 2 AD(LATE-NC-) and 7 AD(LATE-NC+) cases. Here, we captured the pTDP-43 epitopes in the samples (Suppl. Figure 3a, Additional file 1), which were strongly reduced in the non-bound (i.e.: depleted) fraction (Fig. 2e). Importantly, p-tau was also captured when immunoprecipitating pTDP-43 (Suppl. Figure 3b, Additional file 1), hence p-tau levels were also reduced in the depleted fraction of AD(LATE-NC+) cases (Fig. 2e). This suggests that the presence of TDP-43 is necessary to exacerbate tau seeding, probably by increasing p-tau levels through direct interaction and/or stabilization of the aggregates (Fig. 2e, Suppl. Figure 3a-b, Additional file 1). AD(LATE-NC-) cases showed scant levels of p-tau biochemically before depletion (Fig. 2e). Finally, we performed a similar seeding assay as described previously to evaluate tau aggregation when cells are treated with the pTDP-43-depleted samples, compared to their corresponding non-depleted extracts. For this, cells were simultaneously treated with both depleted and non-depleted extracts in the same experiment. We observed that when pTDP-43 was depleted from the extracts, tau aggregation was significantly decreased (*p* = 0.0156, Wilcoxon matched-pairs signed rank test) in AD(LATE-NC+), compared to the corresponding sarkosyl-insoluble samples, i.e.: non-depleted AD(LATE-NC+) (Fig. 2f). The minor seeding effects observed previously with control and AD (LATE-NC-) homogenates were also diminished, although this was not significant (Fig. 2f). Consistently, when treating the tau biosensor cell line with the same p-tau199 concentration (1ng), the seeding effects were similar among AD(LATE-NC-) and AD(LATE-NC+)-treated cells, although cells treated with AD(LATE-NC+) showed slightly higher amounts of tau seeding, but this was not significant (Suppl. Figure 4). This suggests that the observed seeding effects are dependent of p-tau concentration. In turn, it suggests that the presence of TDP-43 pathology impacts p-tau concentration, indirectly impacting tau seeding.

### The presence of LATE-NC is associated with worsened tau seeding in TDP-43^A315T^ mice

**Fig. 3:**
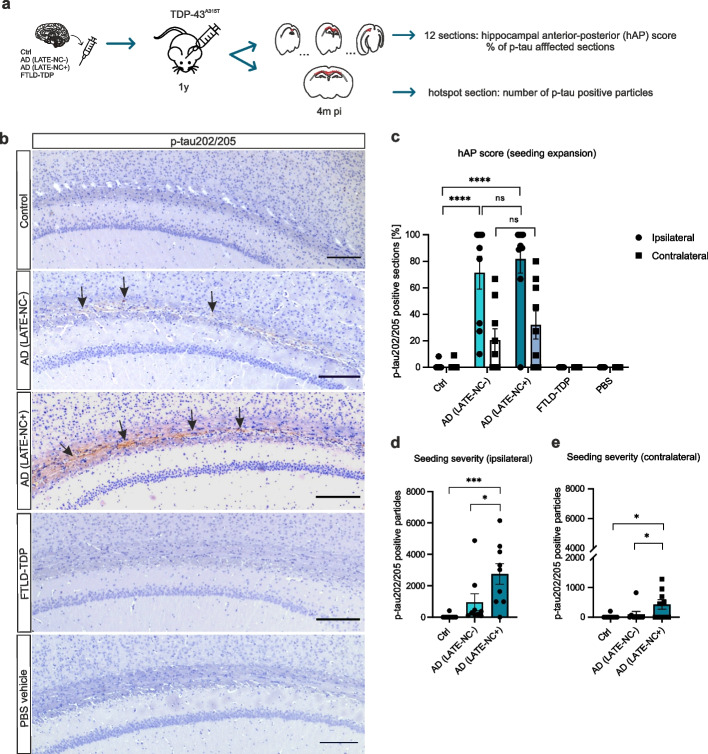
The presence of LATE-NC is associated with p-tau seeding severity in TDP-43^A315T^ mice. **a** Transgenic TDP-43^A315T^ mice were stereotactically injected in the hippocampus at 1 year of age with human brain sarkosyl-insoluble homogenates of 1 aged control, *n* = 9; 1 AD(LATE-NC-), *n* = 10; 1 AD(LATE-NC+), *n* = 9 and 1 FTLD-TDP case, *n* = 7 or with PBS, *n* = 7. Animals were sacrificed 4 months post-injection and their brains were histologically evaluated: the spread of p-tau seeding was evaluated in 12 hippocampal sections covering the whole hippocampus from anterior to posterior hippocampus (hAP score). Among these, a “hotspot” section was selected and the severity of p-tau aggregation was also quantified. **b** P-tau202/204 immunostaining of experimental groups displaying the central white matter band. Extracellular, neuropil p-tau seeds are visible in AD(LATE-NC-) and AD(LATE-NC+) -injected mice, being more pronounced in AD(LATE-NC+)-injected mice (arrows); scale bars = 500µm. **c** Quantification of the hAP score for each experimental groups; average percentages of positive sections (out of 12) are displayed. Animals injected with AD(LATE-NC-) and AD(LATE-NC+) extracts showed a significantly increased hAP score in the ipsilateral and contralateral compared to control-injected animals, pointing to a similar spread of p-tau seeds among both groups. Importantly, AD(LATE-NC+)-injected animals showed an increased number of p-tau202/205-positive particles (i.e.: seeding severity) compared to the AD(LATE-NC-) and control group, in the (**d**) ipsilateral as well as in the (**e**) contralateral. Multiple linear regressions (least squares) were performed to compare the hAP score and the number of p-tau particles among groups. The FTLD-TDP and PBS groups were excluded from both analyses as they showed no p-tau seeding

Next, we investigated the synergy between p-tau and TDP-43 in vivo. First, we analyzed the seeding potential of p-tau in TDP-43^A315T^ transgenic mice, a model that exhibits ubiquitin-positive but TDP-43-negative inclusions, accompanied by spinal motor neuron loss [45]. We performed hippocampal stereotactic injections of sarkosyl-insoluble, patient-derived extracts: 1 control, 1 AD(LATE-NC-), 1 AD(LATE-NC+), 1 FTLD-TDP or PBS-vehicle. The histological and biochemical characterization of the cases used for injections is depicted in Suppl. Figure 5b-d (Additional file 1) and in Tables 2, and 3. The animals were injected at one year of age and sacrificed four months post-injection, which was followed by immunohistochemistry of all experimental groups with a p-tau202/205 antibody. We quantified the expansion of p-tau seeds in the entire hippocampus, from anterior to posterior hippocampus, displayed in percentage of p-tau positive sections (out of 12, designated as hAP score [44]), as well as the local severity, i.e.: the number of p-tau particles in a given “hotspot” section (Fig. 3a). We observed extracellular neuropil staining positive for p-tau in the central white matter band adjacent to the hippocampus, as well as in the corpus callosum in mice that received brain lysates from AD(LATE-NC-) and AD(LATE-NC+) cases, albeit without the presence of tau-positive neurons or NFTs (Fig. 3b, arrows). Animals injected with AD(LATE-NC-) and AD(LATE-NC+) lysates exhibited a higher hAP score when compared to control-injected mice in the ipsilateral (multiple linear regression, *R*^*2*^ = 0.64; *p* < 0.0001, Suppl. Tables 16–17, Additional file 1) but only the AD(LATE-NC+) group showed a difference relative to the control group in the contralateral hemisphere (*R*^*2*^ = 0.25, *p* = 0.0103, Suppl. Table 18, Additional file 1). FTLD-TDP and PBS-injected mice displayed no p-tau seeding (Fig. 3b) and were therefore not included in the analyses. Notably, when quantifying the local p-tau severity, we found that animals injected with AD(LATE-NC+) extracts showed a higher number of p-tau-positive particles in both hemispheres compared to control lysate injected animals (ipsilateral: *R*^*2*^ = 0.39, *p* = 0.0007; contralateral: *R*^*2*^ = 0.26, *p* = 0.0120) and to AD (LATE-NC-)-injected animals (ipsilateral, p = 0.0163; contralateral, *p* = 0.0373) (Fig. 3d-e, Suppl. Tables 19–20, Additional file 1). These results show that AD(LATE-NC+) seeds may impact the severity, although not the propagation speed of p-tau aggregation.

### TDP-43 seeds do not aggregate, but promote its neuronal nuclear loss in TDP-43^A315T^ mice

**Fig. 4:**
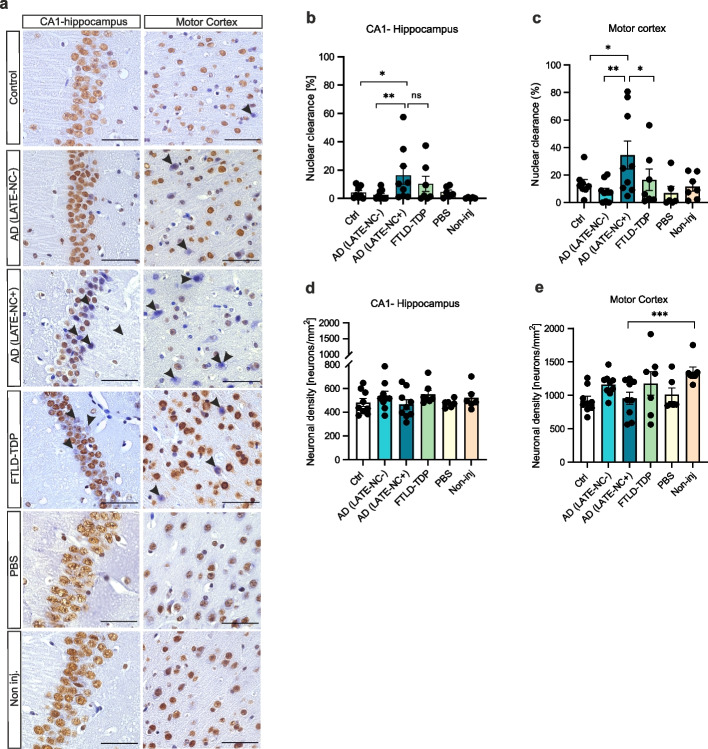
AD(LATE-NC+) seeds impact TDP-43 nuclear clearance in TDP-43^A315T^ mice, but not neuronal loss. **a** Immunohistochemistry with anti- C-terminal TDP-43 (405-414aa.) of mouse experimental groups in CA1-hippocampus and motor cortex. A group of age-matched, non-injected animals was included in this analysis (*n* = 7). AD(LATE-NC+) injected mice display neurons cleared of TDP-43 (arrowheads), scale bars = 50µm. Quantification of the percentage of neurons cleared for C-t TDP-43 of all experimental mouse groups in **(b)** CA1-hippocampus and **(c)** motor cortex. AD(LATE-NC+)-injected animals exhibit higher percentages of nuclear TDP-43 clearance compared to all other groups except FTLD-TDP in the CA1 and to all groups in the motor cortex. Quantification of neuronal density (displayed in neurons/mm^2^) show no relevant differences in the **(d)** CA1 or **(e)** motor cortex. Multiple linear regressions (least squares) were performed to compare TDP-43 nuclear clearance and neuronal density among groups

We also investigated whether the injection of TDP-43 seeds would trigger TDP-43 pathogenesis in TDP-43^A315T^ mice. For this, we analyzed all experimental groups with antibodies against pTDP-43 and non-phosphorylated TDP-43 (405-14aa). No cytoplasmic lesions were observed in either group (Suppl. Figure 6, Additional file 1), however nuclear loss of physiological TDP-43 was apparent (Fig. 4a, arrowheads). We then quantified the percentage of neurons cleared for C-terminal TDP-43 (representing nuclear loss) in the CA1-hippocampus and the motor cortex (ipsilateral) among experimental groups, with the addition of a non-injected group (*n* = 7). In the CA1 region, AD(LATE-NC+) injected animals displayed higher percentages of nuclear clearance compared to AD(LATE-NC-)-injected (*p* = 0.0080), control-injected (*p* = 0.0213), PBS-vehicle (*p* = 0.0393) and non-injected animals (*p* = 0.0059) (multiple linear regression, *R*^*2*^ = 0.24, Fig. 4b, Suppl. Table 21, Additional File 1). Similar results were observed in the motor cortex, where animals injected with AD(LATE-NC+) extracts exhibited more TDP-43 nuclear loss compared to all remaining groups (0.0023 < *p* < 0.0461, *R*^*2*^ = 0.27, Fig. 4c, Suppl. Table 22, Additional file 1). Animals injected with FTLD-TDP homogenates also showed increased nuclear clearance in the hippocampus compared to non-injected animals, however this was not significant (Fig. 4c, Suppl. Table 23, Additional file 1).

We also investigated whether AD(LATE-NC+) seeds would impact neuronal loss in the mouse brain. For this, we quantified the number of neurons per mm^2^ among the different experimental groups (Fig. 4 d-e). No relevant differences were observed, except between AD(LATE-NC+) and non-injected animals in the motor cortex (*p* = 0.0052, Suppl. Tables 24–25, Additional file 1), suggesting that the hippocampal injections, regardless of the injection material, played a minor role in the loss of neurons in TDP-43^A315T^ mice.

## Discussion

In this study, we investigated the impact of TDP-43 pathology (i.e., LATE-NC) in AD, specifically on p-tau pathogenesis. For this, we studied human-post mortem, symptomatic AD cases without and with TDP-43 pathology: AD(LATE-NC-) and AD(LATE-NC+), respectively, and we used in vitro and in vivo approaches to investigate the synergy between TDP-43 and p-tau on a functional level. Our findings extend the current literature suggesting synergistic effects between TDP-43 and tau pathologies by demonstrating that TDP-43 worsens p-tau pathology, resulting in increased p-tau seeding potential.

Specifically, we report that AD(LATE-NC+) cases exhibit higher burdens, as well as higher brain levels of p-tau. We also show that the presence of TDP-43 associates with exacerbated p-tau seeding potential in a tau biosensor cell line, probably by increasing p-tau concentration via direct interaction. Importantly, the injection of AD(LATE-NC+) homogenates in TDP-43^A315T^ mice triggered a more severe p-tau seeding and increased nuclear clearance of physiological TDP-43, compared to animals that received AD(LATE-NC-) and control extracts.

Recently, we have shown that TDP-43 and p-tau physically interact during the progression of AD [15], and that hippocampal p-tau pathology is increased in AD(LATE-NC+) [10]. Moreover, higher tau burdens and higher Braak NFT stages have been observed in AD(LATE-NC+) cases [3]. Consistently, another recent study has shown that the absence of LATE-NC in AD is associated with resilience and resistance to AD pathology [46]. Indeed, an accumulating body of evidence has shown that the co-occurrence of LATE-NC and AD is associated with increased hippocampal atrophy and worse dementia outcomes compared to AD(LATE-NC-), i.e., pure AD [6, 9, 46–48], suggesting that the presence of LATE-NC may lower the threshold for developing clinical symptoms [49]. Here, we speculate that the co-accumulation of TDP-43 aggravates tau pathology, thereby worsening neurodegeneration and ultimately leading to a more severe cognitive decline.

TDP-43 and tau aggregates are well known to co-localize in brains with ADNC [11, 15, 16, 50, 51], and we recently suggested that these pathologies seem to increase in parallel in tau aggregate maturation during the progression of AD [52]. Moreover, several studies have highlighted synergistic effects between these proteins on a molecular level, contributing to neurodegeneration and cell loss. On the one hand, TDP-43 promotes tau mRNA instability and suppresses physiological tau expression [45]. On the other, it has been observed that TDP-43 shifts the ratio of tau repeats by regulating tau mRNA splicing in cell and mouse models [45]. Another study has found that tau tubulin kinases (TTBK1 and TTBK2), which are known to phosphorylate tau in AD, also phosphorylate and co-localize with TDP-43 in FTLD cases and other animal models [53]. Additionally, both tau and TDP-43 have been implicated in the disruption of nuclear-cytoplasmic transport in AD and ALS and FTLD, respectively [54, 55], therefore it is possible that these proteins are involved in this mechanism in cases with both ADNC and LATE-NC. Finally, recent studies have shown that TDP-43 promotes p-tau accumulation and tau-driven neurotoxicity in an animal model [18] and that TDP-43 may serve as a templating agent to stabilize tau aggregates [56]. On the other hand, tau seems to play a less robust role in modulating TDP-43 aggregation and toxicity [18]. Taken together, these data point to a biological link between tau and TDP-43, which seem to share a common pathological cascade, probably also sharing genetic pleiotropy, i.e.: APOE ε4 [9, 13, 57], even though the molecular underpinnings of this synergy are not yet fully understood.

In this study, we show that AD(LATE-NC+) cases show higher p-tau burdens (tangles and pretangles) as well as increased p-tau199 levels compared to AD(LATE-NC-) cases in the entorhinal and frontal cortices. Interestingly, the levels of physiological TDP-43 were decreased in the entorhinal cortex of FTLD-TDP cases compared to AD(LATE-NC-), AD(LATE-NC+) and controls. We postulate that this points to the loss of function of physiological TDP-43 in these cases, which has been reported in other models [58, 59] and is likely associated with neuronal death.

Because AD(LATE-NC+) cases also have higher p-tau concentrations, it was not unexpected that the p-tau seeding observed in cells and animals treated with AD(LATE-NC+) extracts was also exacerbated. Indeed, when treating cells with similar p-tau concentrations, the seeding effects were similar. However, after immunoprecipitating pTDP-43 and treating the tau biosensor cell line with pTDP-depleted sarkosyl-insoluble homogenates from AD(LATE-NC+) cases, p-tau seeding decreased dramatically. This suggests that the presence of TDP-43 is linked and somewhat necessary to the acceleration of p-tau seeding. We hypothesize that TDP-43 may help increasing p-tau concentration in the brain, indirectly facilitating tau-driven seeding.

The anatomical spread and propagation speed of p-tau seeds in the entire mouse hippocampus (hAP score) was similar upon the injection of both AD(LATE-NC-) and AD(LATE-NC+) extracts. However, animals injected with AD(LATE-NC+) material exhibited a more severe local p-tau seeding, compared to animals injected with AD (LATE-NC-) homogenates. This observation suggests that TDP-43 pathology is more important for increasing local p-tau severity, rather than promoting the spatial spreading of tau aggregates. Accordingly, even though TDP-43 distribution in the AD brain seems to recapitulate that of tau [25], it is likely that TDP-43 proteinopathy in AD accumulates after tau pathology is established, exacerbating its severity and leading to increased neuronal loss and faster rates of hippocampal atrophy [10, 47]. For these reasons, we postulate that the synergy between tau and TDP-43 may be more relevant in the limbic-predominant and typical subtypes of AD [60], as TDP-43 aggregates rarely spread to the cortical areas in AD [5, 25].

Unexpectedly, we observed no pretangles, NFTs or dystrophic neurites on the injection site, i.e.: hippocampus, or neighboring areas. One reason for this might be the fact that these mice were analyzed four months post-injection, which could mean that tau aggregates could require a longer period to form in the mouse brain. Accordingly, another study has investigated the seeding potential of AD-derived tau seeds in non-transgenic mice in order to recapitulate sporadic AD, which only significantly increased tau seeding nine months post-injection [61]. Moreover, other tau species such as soluble monomers and oligomers, but not sarkosyl-insoluble tau species, could be more prone to cause tau seeding and spreading [62, 63].

Although we did not observe the presence of TDP-43 aggregates in TDP-43^A315T^ mice even four months after injection of human seeds, there was an augmented loss of physiological nuclear TDP-43. Indeed, several TDP-43 mouse models do not display aggregated TDP-43, despite the presence of symptoms at time of death as well as neurodegeneration signs post-mortem [64–68]. This suggests that aggregate formation might not be necessary to initiate TDP-43-driven neurodegeneration, and that it could rather result from the loss of physiological, nuclear TDP-43. Accordingly, we report that the injection of AD(LATE-NC+) homogenates promoted a significant increase in the number of neurons cleared for nuclear TDP-43 in the hippocampus and motor cortex of TDP-43^A315T^ mice. The loss of TDP-43 function from the nucleus upon a stressful and/or pathological stimulus has been previously reported in the literature as a possible mechanism for TDP-43 pathogenesis [4, 58]. We and others have hypothesized that this could possibly represent an early step in disease pathogenesis where TDP-43 aggregates are a pathological hallmark, i.e.: ALS/FTLD spectrum and AD [69–71]. Moreover, nuclear loss of TDP-43 has been previously detected in this model, being occasionally present in neurons with ubiquitin-positive but TDP-43-negative lesions [39]. Our results further suggest that the loss of TDP-43 occurs before the appearance of abnormal TDP-43 lesions. The data also indicate that the loss of nuclear TDP-43 occurs before neuronal cell death, as we found no significant differences in neuronal loss in the motor cortex between the experimental groups. Consistently, we and others have previously observed neuronal loss in spinal cord and motor cortex of late-stage TDP-43^A315T^ mice [39, 64]. Nevertheless, novel models that recapitulate both tau and LATE-NC proteinopathies will be crucial to further elucidate tau and TDP-43 pathogeneses and synergistic effects in vivo, as well as determine how TDP-43 enhances tau pathology, or vice versa [8]. Studies addressing the pathological interplay between Aβ, tau and TDP-43 will also be of interest.

A confounding factor that may also influence pathogenesis, seeding and the clinical phenotype in AD(LATE-NC+) cases is the presence of additional co-pathologies, such as α-synuclein. Importantly, both p-tau and TDP-43 were shown to co-localize with α-synuclein in AD [72, 73], therefore it is likely that this binding partner also contributes synergistically to exacerbating AD. In our cohort, the mean Braak LBD stage among both AD groups was similar, with AD(LATE-NC+) cases showing a slightly higher mean (1.4) compared to AD(LATE-NC-) cases (0.25), so it is likely that α-synuclein plays only a minor role in exacerbating p-tau pathology in AD.

One limitation of this study is the low number of AD (LATE-NC-) cases in our cohort, i.e., without any detectable LATE-NC. This is probably due to stringent criteria employed in our study to group the cases, as we considered a case positive for LATE-NC if a single TDP-43 lesion was found in one or more of the following regions: amygdala, posterior hippocampus, and middle-frontal cortex. Additionally, this can also be explained due to the use of a hospital-based cohort, which can be enriched for co-pathologies [74]. However, the purpose of this study was to address the differences in p-tau pathology in demented individuals and to perform functional studies using patient-derived material. Another limitation is that this study focused on the “younger-old”, especially the controls, with a mean age of 64.7. This was probably because hospital-based cohorts tend to have younger ages at death. Additionally, the criteria for selecting controls included the absence of Aβ plaques (Aβ phase 0), excluding a high number of cases with advanced age [75]. This may limit the interpretation to the general population.

Additionally, the mouse model used in this study does not necessarily recapitulate TDP-43 pathology occurring in AD, as LATE-NC models are not yet available. A final limitation is the fact that the impact of Aβ pathology on tau and TDP-43 pathologies was not investigated in this study. However, there were no significant differences in Aβ phases among AD cases (Table 1), so we believe that it’s unlikely that Aβ plays a major role in TDP-43/tau synergy.

Despite these limitations, we demonstrated that the presence of TDP-43 pathology is associated with worsened AD-related tau pathology in the human AD brain and in two different models with a tau or a TDP-43 background. Further, we showed that in the absence of pTDP-43, p-tau expression was decreased, as well as p-tau seeding. Future experimental studies should focus on preventing this interaction and investigate the impact of p-tau and TDP-43 proteinopathies on cognition.

**Fig. 5:**
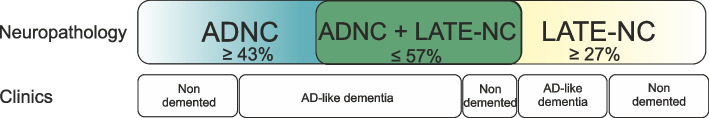
Most dementia cases exhibit either AD pathology, LATE pathology or a combination of the two. The majority of symptomatic AD cases present comorbid TDP-43 pathology, i.e., LATE-NC alongside Aβ plaques and tau tangles (up to 57% of AD cases [3, 46, 81]). “Pure” LATE-NC cases with no detectable Aβ plaques also exist (27% of older adults [7]), which can be demented or non-demented

TDP-43 pathology, i.e., LATE-NC is known to occur in brains with little or no ADNC [7, 76]. Consistently, pure AD cases are also common, although such cases seem to present lower levels of AD pathology [46] and disease severity, when compared to AD cases with LATE-NC [3, 48]. Of note, the co-occurrence of TDP-43 and tau pathologies is not unique to AD, occurring also in primary age-related tauopathy (PART) and FTLD-TDP cases [42, 77–79], however this synergy appears to play a much more relevant role in AD. Because LATE-NC co-occurs in most clinical AD cases alongside Aβ plaques and tau NFTs [3, 7] (Fig. 5), this study has an impact in the understanding of TDP-43 pathogenesis in AD and LATE, which account for the majority of dementia cases worldwide [2, 6, 80].

## Conclusions

Taken together, our findings extend the current knowledge suggesting an association between TDP-43 and tau pathologies, by demonstrating their functional synergy, resulting in increased p-tau seeding potential. Moreover, this study stresses the relevance of comorbidities that contribute to dementia, which are more often the rule than the exception [82, 83]. Our results further highlight that AD patients with comorbid LATE-NC should be distinguished in a clinical setting, as the treatment required for these patients might differ from that of pure AD [8], especially when targeting tau. Thus, these data urge for the development of biomarkers that detect TDP-43 pathology during life, in order to properly stratify demented individuals with co-morbid LATE-NC.

### Supplementary Information

**Additional file 1.** Supplementary material including supplementary Figures and Tables.

## Data Availability

The anonymized datasets used and/or analyzed during the current study are stored in UZ/KU-Leuven network drives and available from the corresponding author on reasonable request.

## Abbreviations

AD: Alzheimer's Disease
ADNC: Alzheimer's Disease neuropathological changes
AD(LATE-NC-): Alzheimer's Disease cases without TDP-43 pathology (LATE-NC)
AD(LATE-NC+): Alzheimer's Disease cases with TDP-43 pathology (LATE-NC)
ALS: Amyotrohophic Lateral Sclerosis
APOE ε4: Apolipoprotein E4
BCA: Bicinchoninic acid assay
BSA: Bovine serum albumin
CA1: Cornu ammonis 1
CDR: Clinical dementia rating
CFP: Cyan Fluorescent Protein
CO_2_: Carbon dioxide
DAB: Diaminobenzidine
DAPI: 4',6-diamidino-2-phenylindole
DMEM: Dulbecco's Modified Eagle Medium
DNA: Deoxyribonucleic acid
ELISA: Enzyme-linked immunoassay
FBS: Fetal bovine serum
FRET: Fluorescence resonance energy transfer
FTLD-TDP: Frontotemporal lobar degeneration with TDP-43 inclusions
LATE: Limbic-predominant age-related TDP-43 encephalopathy
LATE-NC: Limbic-predominant age-related TDP-43 encephalopathy neuropathological changes
LBD: Lewy Body Disease
NA: Non-applicable
NCI: Neuronal cytoplasmic inclusion
NFT: Neuronal fibrillary tangle
NIA-AA: National Institute on Aging - Alzheimer's Association
PBS: Phosphate buffer saline
RNA: Ribonucleic acid
SDS: Sodium Dodecyl Sulfate
TDP-43: Transactive response DNA binding protein of 43 kDa
YFP: Yellow Fluorescent Protein
