# Supplementary material for: TDP-43 pathology is associated with increased tau burdens and seeding
Source: Mol Neurodegener. 2023 Sep 30;18:71. doi: 10.1186/s13024-023-00653-0 (PMC10544192; doi:10.1186/s13024-023-00653-0)
Supplement: Supplementary file 1 — Additional file 1. Supplementary material including supplementary Figures and Tables. [file 13024_2023_653_MOESM1_ESM.docx]

**Additional File 1:**

**TDP-43 pathology is associated with increased tau burdens and seeding**

Sandra O. Tomé ^1,2^ *, Grigoria Tsaka ^1,2,3,4^, Alicja Ronisz^1,2^, Simona Ospitalieri^1,2^, Klara Gawor^1,2^, Luis Aragão Gomes^1,2^, Markus Otto^5,6^, Christine A.F. von Arnim^5,7^, Philip Van Damme^2,8,12^, Ludo Van Den Bosch^2,8^, Estifanos Ghebremedhin^9^, Celeste Laureyssen^10,11^, Kristel Sleegers^10,11^, Rik Vandenberghe^2,12,13^, Frederic Rousseau^3,4^, Joost Schymkowitz^3,4^, Dietmar Rudolf Thal^1,2,14^

^1^Laboratory of Neuropathology – Department of Imaging and Pathology, KU Leuven, Leuven, Belgium.

^2^Leuven Brain Institute, KU Leuven, Leuven, Belgium.

^3^ Switch Laboratory, VIB-KU Leuven Center for Brain & Disease Research, Leuven, Belgium

^4^ Department of Cellular and Molecular Medicine, KU Leuven, Leuven, Belgium

^5^Department of Neurology, University of Ulm, Ulm, Germany

^6^Department of Neurology, University of Halle, Halle, Germany

^7^Department of Geriatrics, University Medical Center Göttingen, Göttingen, Germany

^8^Laboratory for Neurobiology – VIB-KU Leuven, Leuven, Belgium

^9^Institute for Clinical Neuroanatomy – Johann Wolfgang Goethe University, Frankfurt am

Main, Germany

^10^Complex Genetics of Alzheimer’s Disease group, VIB-University of Antwerp Center for Molecular Neurology, Antwerp, Belgium

^11^Department of Biomedical Sciences, University of Antwerp, Antwerp, Belgium

^12^Department of Neurology, UZ Leuven, Leuven, Belgium

^13^Laboratory of Experimental Neurology - Department of Neurosciences, KU Leuven, Leuven, Belgium

^14^Department of Pathology, UZ Leuven, Leuven, Belgium.

* Corresponding author

**Supplementary figures:**


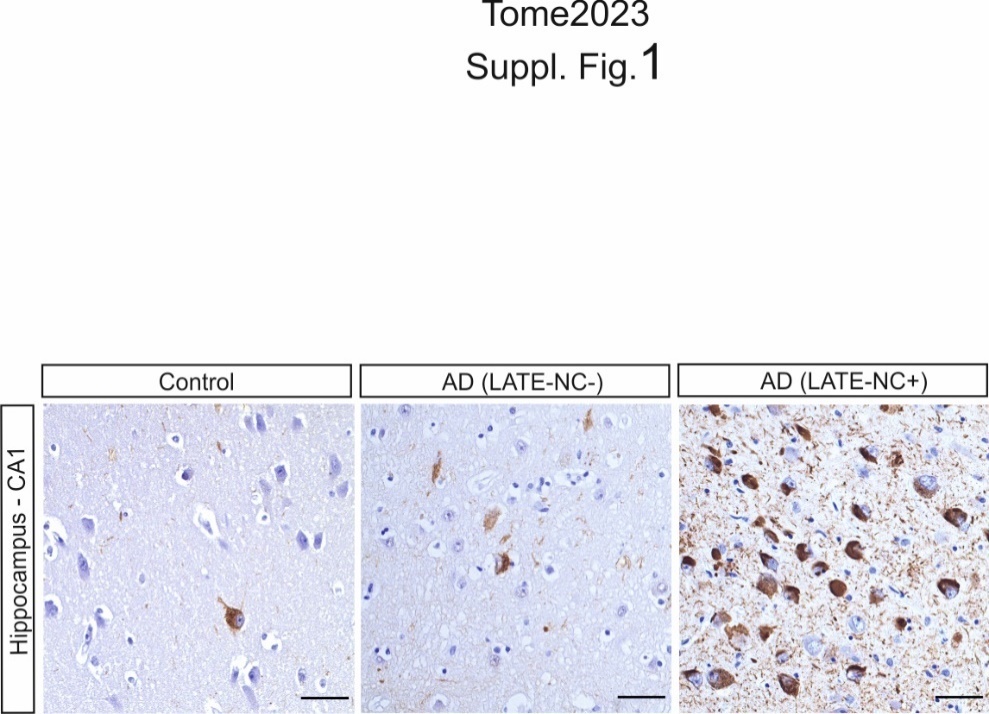


**Supplementary Figure 1 – p-tau181 immunostaining of a control, AD(LATE-NC-) and AD(LATE-NC+) cases.** P-tau181 immunostaining (mouse monoclonal, clone AT270, 1:5000; ThermoFisher Scientific) shows similar results to p-tau202/205 (ThermoFisher Scientific), n=5 per group. Scale bars = 50µm.


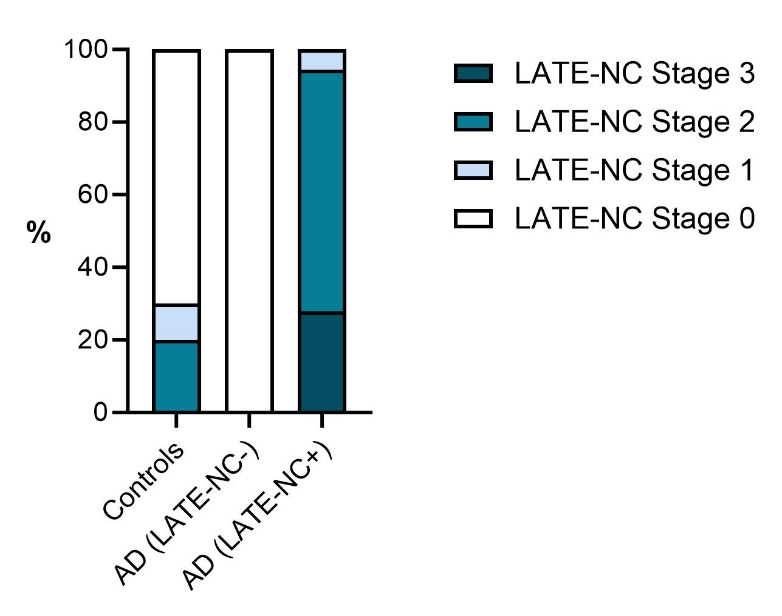


**Supplementary Figure 2 – LATE-NC stages distribution among the study cohort.** Controls, 70% LATE-NC stage 0, 10% LATE-NC stage 1, 20% LATE-NC stage 2. AD(LATE-NC+), 5.6% LATE-NC Stage 1, 66.6% LATE-NC stage 2, 20% LATE-NC stage 3. N=93.


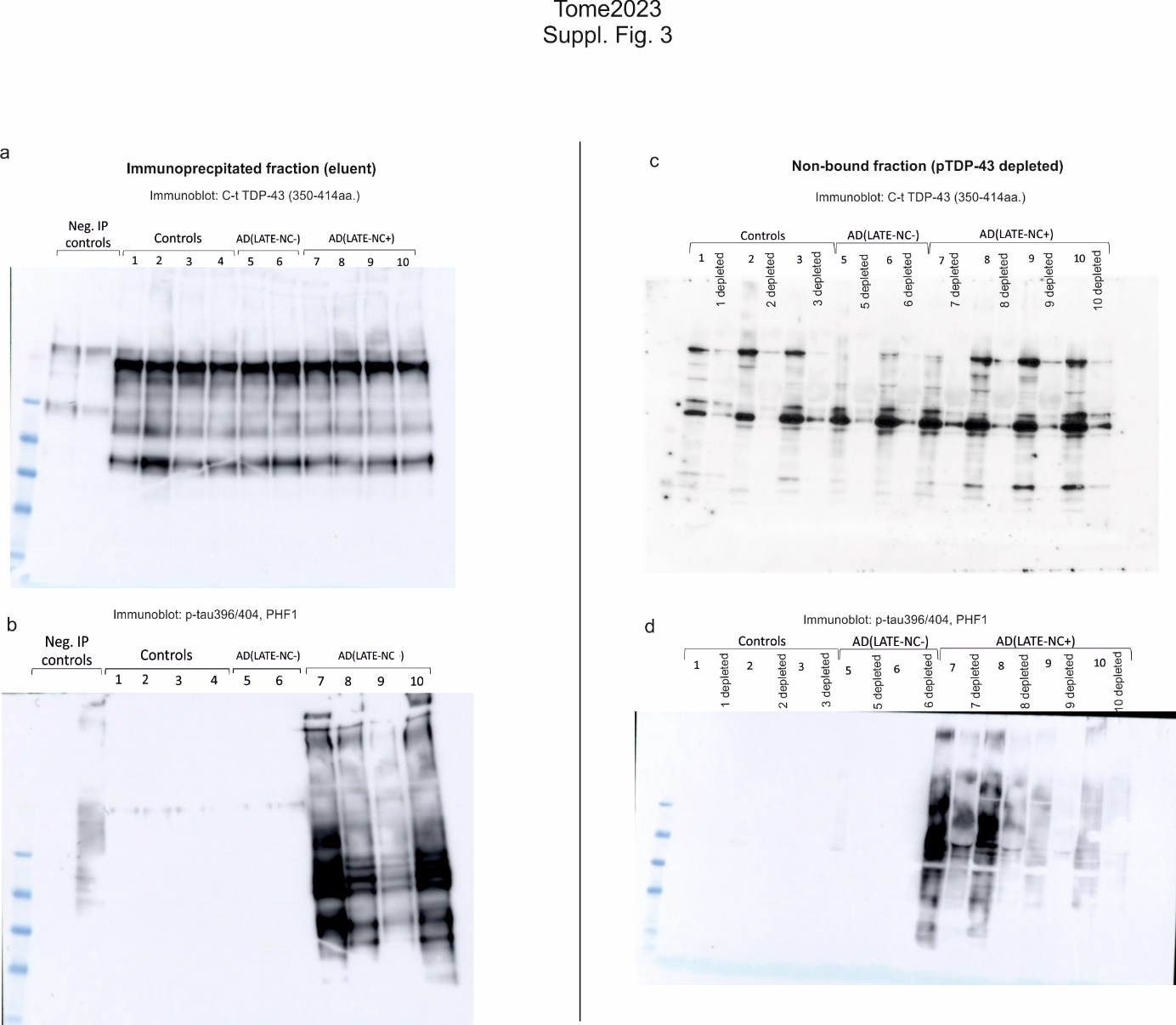


**Supplementary Figure 3 – pTDP-43 immunoprecipitation reduces p-tau levels in the non-bound fraction. (a)** Sarkosyl-insoluble homogenates from 4 controls, 2 AD(LATE-NC-) and 4 AD(LATE-NC+) cases were immunoprecipitated with pTDP-43 (409/410, #22309-1-AP, Proteintech) and immunoblotted with a C-terminal TDP-43 antibody (350-414aa., ThermoFisher Scientific)**. (b)** The same membrane was probed with p-tau396/404, showing that p-tau binds to pTDP-43. The full western blot membranes from the non-bound fractions (pTDP-43 depleted fractions) as shown in Fig. 2e are shown in **(c-d)**.


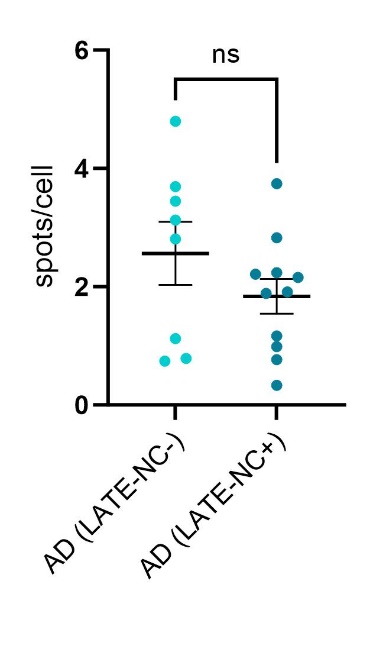


**Supplementary Figure 4 – Increased seeding effect is mostly dependent of p-tau concentration.** When exposing the tau biosensor cell line to the same p-tau concentration (1 ng), cells treated with frontal cortex homogenates from AD(LATE-NC-) and AD(LATE-NC+) cases showed similar amounts of seeding, suggesting an indirect role for TDP-43 pathology in exacerbating p-tau seeding. AD(LATE-NC-), n=8; AD(LATE-NC+), n=11. Unpaired, two-tailed t- test was performed (*p* = 0.267) and means ± SEM are shown.


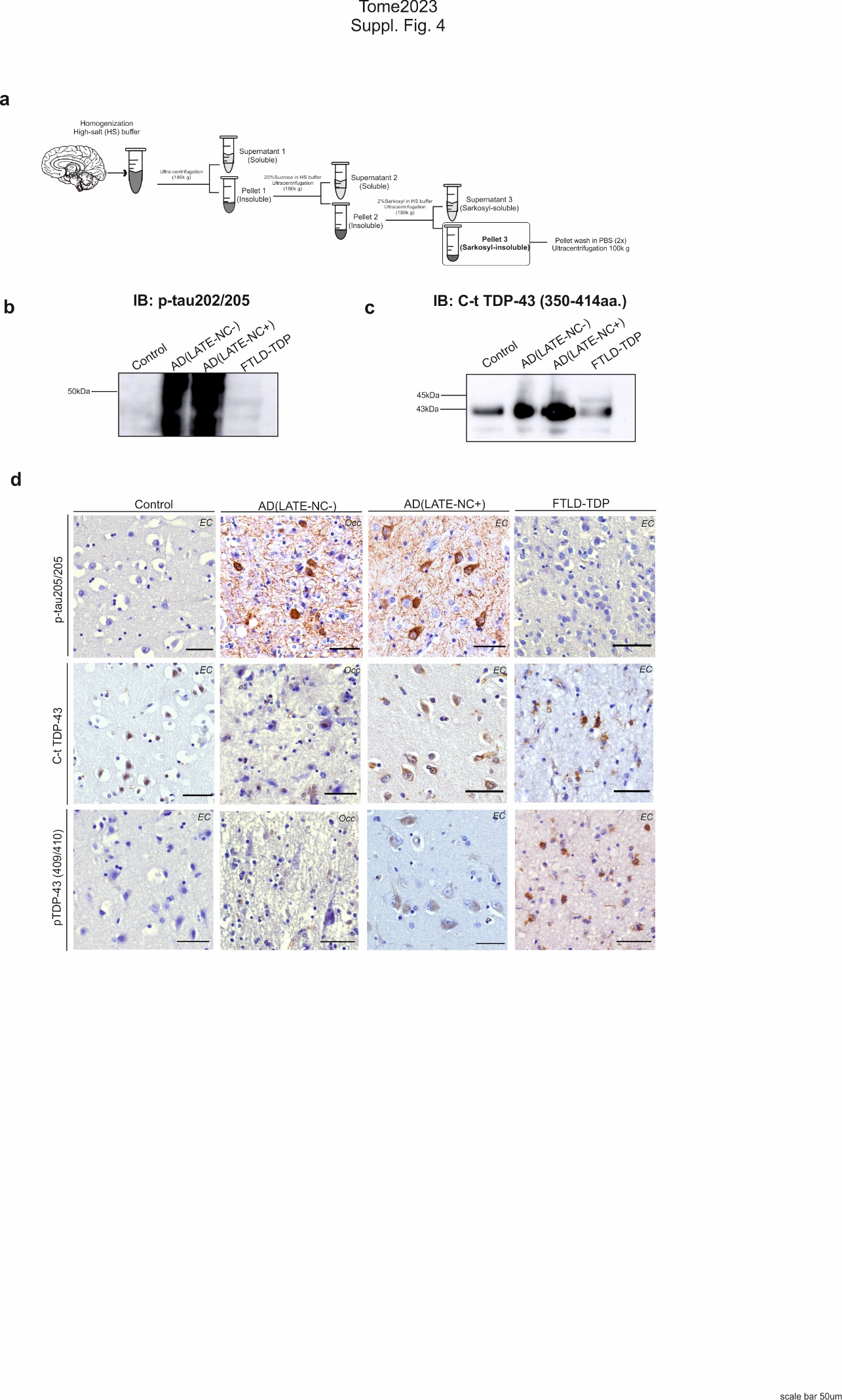
**Supplementary Figure 5 – Characterization of patient-derived homogenates used for stereotactic injection. (a)** Protein extraction protocol used to obtain sarkosyl-insoluble extracts. Western blot analysis of **(b)** p-tau396/404 (mouse monoclonal, 1:2000, gift from Peter Davies), and **(c)** C-terminal TDP-43 (350-414aa., rabbit polyclonal, 1:1000, ThermoFisher Scientific) reveal increased levels of p-tau and TDP-43 in AD cases. A 45kDa TDP-43 band is visible in the FTLD-TDP case. **(d)** Immunohistochemistry of brain regions for mouse injection with antibodies against p-tauS202/205 (mouse monoclonal, 1:1000, ThermoFisher Scientific), C-t TDP-43 (405-414aa., rabbit polyclonal, 1:5000, Cosmobio) and pTDP-43 (409/410, rabbit polyclonal, 1:5000 Cosmobio). Only AD cases (occipital and entorhinal) show p-tau lesions. AD(LATE-NC+) and FTLD-TDP case shows cytoplasmic TDP-43 inclusions and dystrophic neurites positive for C-t TDP-43 and pTDP-43 antibodies. Scale bars=50µm.


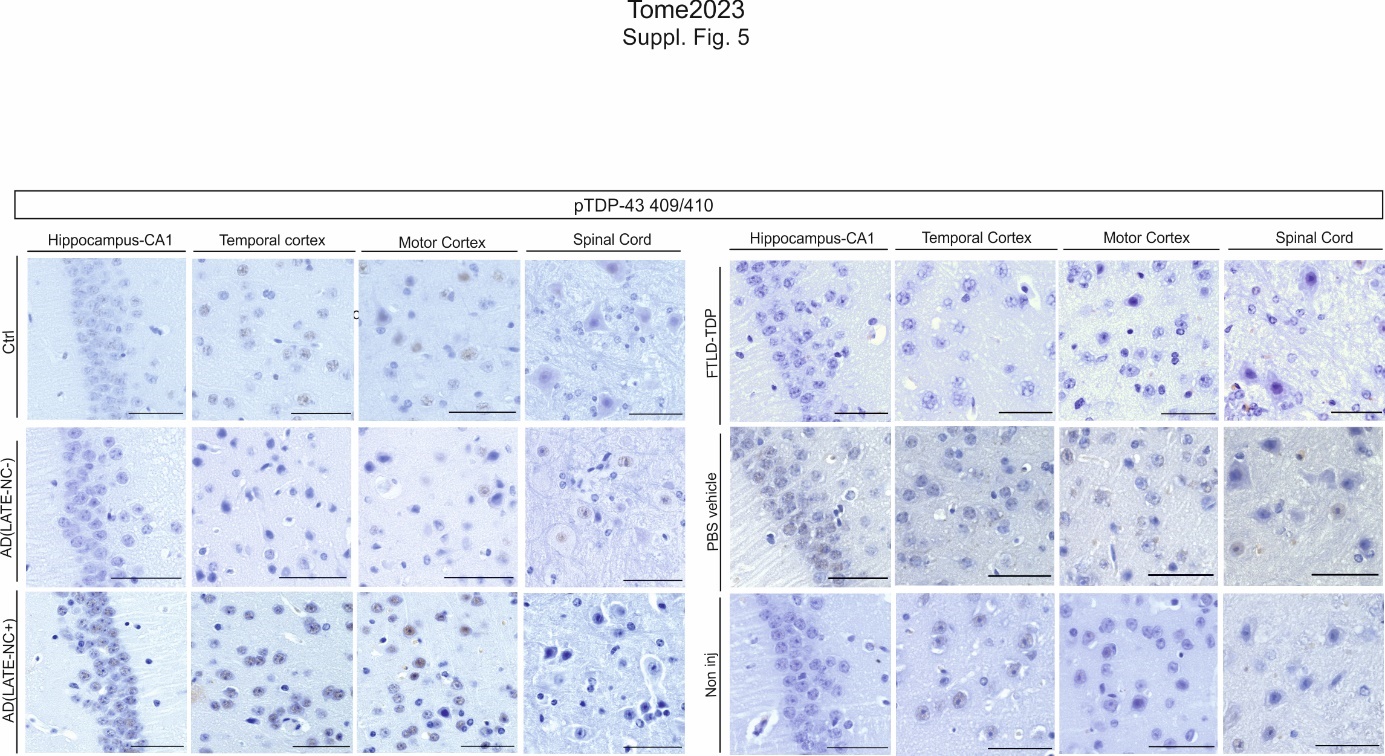
**Supplementary Figure 6 - TDP-43 does not seed in the TDP-43 mouse brain.** Immunohistological overview of mouse experimental groups with a pTDP-43 antibody (409/410, rabbit polyclonal, 1:5000 Cosmobio) show no TDP-43 pathology in different brain regions or spinal cord. Scale bars=50µm.

**Supplementary tables:**

**Supplementary Table 1 –** Multiple linear regression, using p-tau202/205 pathology (CA1) as dependent variable and neuropathological groups, age at death, sex and APOE ε4 status as independent variables (n=98).

| Independent variables | Estimate | Standard error | 95% CI (asymptotic) | \|t\| | P value |
| --- | --- | --- | --- | --- | --- |
| Controls | -20,72 | 9,408 | -39,47 to -1,969 | 2,202 | 0,0308 |
| AD (LATE-NC+) | 22,2 | 7,933 | 6,386 to 38,01 | 2,798 | **0,0066** |
| Age | 0,08599 | 0,2659 | -0,4439 to 0,6158 | 0,3234 | 0,7473 |
| Sex | 1,692 | 5,383 | -9,036 to 12,42 | 0,3143 | 0,7542 |
| APOE ε4 | 8,677 | 5,723 | -2,728 to 20,08 | 1,516 | 0,1338 |
| Reference group: AD (LATE-NC-) | | | | | |
| Degrees of Freedom: 73 | | | | | |
| R squared: 0,5139 | | | | | |

**Supplementary Table 2 –** Multiple linear regression, using p-tau202/205 pathology (frontal cortex) as dependent variable and neuropathological groups, age at death, sex and APOE ε4 status independent variables (n=98).

| Independent variables | Estimate | Standard error | 95% CI (asymptotic) | \|t\| | P value |
| --- | --- | --- | --- | --- | --- |
| Controls | -12,87 | 3,803 | -20,45 to -5,288 | 3,383 | **0,0012** |
| AD (LATE-NC+) | 8,511 | 3,205 | 2,123 to 14,90 | 2,655 | **0,0097** |
| Age | -0,4677 | 0,1075 | -0,6819 to -0,2534 | 4,351 | **<0,0001** |
| Sex | -0,3432 | 2,175 | -4,678 to 3,992 | 0,1578 | 0,8751 |
| APOE ε4 | -0,6175 | 2,312 | -5,225 to 3,990 | 0,2671 | 0,7902 |
| Reference group: AD (LATE-NC-) | | | | | |
| Degrees of Freedom: 73 | | | | | |
| R squared: 0,4403 | | | | | |

**Supplementary Table 3 –** Multiple linear regression, using p-tau202/205 pathology (CA1) as dependent variable and neuropathological groups, age at death, sex and APOE ε4 status as independent variables (n=98).

| Independent variables | Estimate | Standard error | 95% CI (asymptotic) | \|t\| | P value |
| --- | --- | --- | --- | --- | --- |
| AD (LATE-NC-) | -1,605 | 4,588 | -10,75 to 7,539 | 0,3498 | 0,7275 |
| AD (LATE-NC+) | 13,98 | 3,724 | 6,557 to 21,40 | 3,754 | **0,0003** |
| Age | 0,08155 | 0,13 | -0,1776 to 0,3407 | 0,6272 | 0,5325 |
| Sex | 1,827 | 2,632 | -3,419 to 7,072 | 0,6941 | 0,4898 |
| APOE ε4 | 5,172 | 2,8 | -0,4086 to 10,75 | 1,847 | 0,0688 |
| Reference group: Controls | | | | | |
| Degrees of Freedom: 73 | | | | | |
| R squared: 0,4237 | | | | | |

**Supplementary Table 4 –** Multiple linear regression, using p-tau202/205 pathology (frontal cortex) as dependent variable and neuropathological groups, age at death, sex and APOE ε4 status as independent variables (n=98).

| Independent variables | Estimate | Standard error | 95% CI (asymptotic) | \|t\| | P value |
| --- | --- | --- | --- | --- | --- |
| AD (LATE-NC-) | 12,87 | 3,803 | 5,288 to 20,45 | 3,383 | **0,0012** |
| AD (LATE-NC+) | 21,38 | 3,092 | 15,22 to 27,54 | 6,913 | **<0,0001** |
| Age | -0,4677 | 0,1075 | -0,6819 to -0,2534 | 4,351 | **<0,0001** |
| Sex | -0,3432 | 2,175 | -4,678 to 3,992 | 0,1578 | 0,8751 |
| APOE ε4 | -0,6175 | 2,312 | -5,225 to 3,990 | 0,2671 | 0,7902 |
| Reference group: Controls | | | | | |
| Degrees of Freedom: 73 | | | | | |
| R squared: 0,4403 | | | | | |

**Supplementary Table 5 –** Multiple linear regression, using p-tau199 brain levels (entorhinal cortex) as dependent variable and neuropathological groups as independent variables (n=34).

| Independent variables | Estimate | Standard error | 95% CI (asymptotic) | \|t\| | P value |
| --- | --- | --- | --- | --- | --- |
| Controls | -61217 | 6517 | -74526 to -47907 | 9,393 | **<0,0001** |
| AD (LATE-NC-) | -46645 | 6931 | -60799 to -32491 | 6,73 | **<0,0001** |
| FTLD-TDP | -60648 | 8045 | -77078 to -44219 | 7,539 | **<0,0001** |
| Reference group: AD (LATE-NC+) | | | | | |
| Degrees of Freedom: 30 | | | | | |
| R squared: 0,7855 | | | | | |

**Supplementary Table 6 –** Multiple linear regression, using p-tau199 brain levels (frontal cortex) as dependent variable and neuropathological groups as independent variables (n=34).

| Independent variables | Estimate | Standard error | 95% CI (asymptotic) | \|t\| | P value |
| --- | --- | --- | --- | --- | --- |
| Controls | -47598 | 5602 | -59040 to -36157 | 8,496 | **<0,0001** |
| AD (LATE-NC-) | -45437 | 5958 | -57604 to -33269 | 7,627 | **<0,0001** |
| FTLD-TDP | -47540 | 6915 | -61663 to -33417 | 6,874 | **<0,0001** |
| Reference group: AD (LATE-NC+) | | | | | |
| Degrees of Freedom: 30 | | | | | |
| R squared: 0,7682 | | | | | |

**Supplementary Table 7 –** Multiple linear regression, using p-tau199 brain levels (entorhinal cortex) as dependent variable and neuropathological groups as independent variables (n=34).

| Independent variables | Estimate | Standard error | 95% CI (asymptotic) | \|t\| | P value |
| --- | --- | --- | --- | --- | --- |
| AD (LATE-NC-) | 14572 | 7075 | 123,1 to 29021 | 2,06 | 0,0482 |
| AD (LATE-NC+) | 61217 | 6517 | 47907 to 74526 | 9,393 | **<0,0001** |
| FTLD-TDP | 568,6 | 8169 | -16116 to 17253 | 0,0696 | 0,945 |
| Reference group: Controls | | | | | |
| Degrees of Freedom: 30 | | | | | |
| R squared: 0,7855 | | | | | |

**Supplementary Table 8 –** Multiple linear regression, using p-tau199 brain levels (frontal cortex) as dependent variable and neuropathological groups as independent variables (n=29).

| Independent variables | Estimate | Standard error | 95% CI (asymptotic) | \|t\| | P value |
| --- | --- | --- | --- | --- | --- |
| AD (LATE-NC-) | 2162 | 6082 | -10259 to 14582 | 0,3554 | 0,7248 |
| AD (LATE-NC+) | 47598 | 5602 | 36157 to 59040 | 8,496 | **<0,0001** |
| FTLD-TDP | 58,43 | 7023 | -14284 to 14401 | 0,008319 | 0,9934 |
| Reference group: Controls | | | | | |
| Degrees of Freedom: 30 | | | | | |
| R squared: 0,7862 | | | | | |

**Supplementary Table 9 –** Multiple linear regression, using total TDP-43 brain levels (entorhinal cortex) as dependent variable and neuropathological groups as independent variables (n=34).

| Independent variables | Estimate | Standard error | 95% CI (asymptotic) | \|t\| | P value |
| --- | --- | --- | --- | --- | --- |
| Controls | -34,87 | 21,49 | -78,82 to 9,086 | 1,622 | 0,1155 |
| AD (LATE-NC-) | 9,653 | 22,85 | -37,09 to 56,40 | 0,4224 | 0,6759 |
| FTLD-TDP | -93,92 | 28,72 | -152,6 to -35,18 | 3,27 | **0,0028** |
| Reference group: AD (LATE-NC+) | | | | | |
| Degrees of Freedom: 29 | | | | | |
| R squared: 0,3360 | | | | | |

**Supplementary Table 10 –** Multiple linear regression, using total TDP-43 brain levels (frontal cortex) as dependent variable and neuropathological groups as independent variables (n=34).

| Independent variables | Estimate | Standard error | 95% CI (asymptotic) | \|t\| | P value |
| --- | --- | --- | --- | --- | --- |
| Controls | -6,574 | 23,91 | -55,40 to 42,25 | 0,275 | 0,7852 |
| AD (LATE-NC-) | -17,15 | 25,43 | -69,08 to 34,77 | 0,6746 | 0,5051 |
| FTLD-TDP | -35,68 | 29,51 | -95,95 to 24,59 | 1,209 | 0,2361 |
| Reference group: AD (LATE-NC+) | | | | | |
| Degrees of Freedom: 30 | | | | | |
| R squared: 0,05147 | | | | | |

**Supplementary Table 11 –** Multiple linear regression, using total TDP-43 brain levels (entorhinal cortex) as dependent variable and neuropathological groups as independent variables (n=34).

| Independent variables | Estimate | Standard error | 95% CI (asymptotic) | \|t\| | P value |
| --- | --- | --- | --- | --- | --- |
| AD (LATE-NC-) | 44,52 | 23,33 | -3,196 to 92,24 | 1,908 | 0,0663 |
| AD (LATE-NC+) | 34,87 | 21,49 | -9,086 to 78,82 | 1,622 | 0,1155 |
| FTLD-TDP | -59,05 | 29,1 | -118,6 to 0,4637 | 2,029 | 0,0517 |
| Reference group: Controls | | | | | |
| Degrees of Freedom: 29 | | | | | |
| R squared: 0,3360 | | | | | |

**Supplementary Table 12 –** Multiple linear regression, using total TDP-43 brain levels (frontal cortex) as dependent variable and neuropathological groups as independent variables (n=34).

| Independent variables | Estimate | Standard error | 95% CI (asymptotic) | \|t\| | P value |
| --- | --- | --- | --- | --- | --- |
| AD (LATE-NC-) | -10,58 | 25,95 | -63,58 to 42,43 | 0,4075 | 0,6865 |
| AD (LATE-NC+) | 6,574 | 23,91 | -42,25 to 55,40 | 0,275 | 0,7852 |
| FTLD-TDP | -29,11 | 29,97 | -90,31 to 32,10 | 0,9711 | 0,3392 |
| Reference group: Controls | | | | | |
| Degrees of Freedom: 30 | | | | | |
| R squared: 0,05147 | | | | | |

**Supplementary Table 13 –** P-values corresponding to Spearman r partial correlation analysis shown in Fig. 1j (n=98).

|  | 1 | 2 | 3 | 4 | 5 | 6 | 7 | 8 | 9 | 10 | 11 | 12 | 13 | 14 |
| --- | --- | --- | --- | --- | --- | --- | --- | --- | --- | --- | --- | --- | --- | --- |
| 1.CA1 neuronal density |  |  |  |  |  |  |  |  |  |  |  |  |  |  |
| 2.Frontal cx neuronal density | **0,028** |  |  |  |  |  |  |  |  |  |  |  |  |  |
| 3.CA1 pTDP-43 pathology | **0,002** | 0,664 |  |  |  |  |  |  |  |  |  |  |  |  |
| 4.CA1 p-tau pathology | **< 0.001** | 0,546 | **< 0.001** |  |  |  |  |  |  |  |  |  |  |  |
| 5.Frontal cx p-tau pathology | **0,001** | 0,456 | **< 0.001** | **< 0.001** |  |  |  |  |  |  |  |  |  |  |
| 6.Age at death | 0,317 | 0,069 | **0,003** | **0,001** | 0,412 |  |  |  |  |  |  |  |  |  |
| 7.Sex | 0,135 | 0,453 | 0,164 | **0,035** | 0,068 | **0,005** |  |  |  |  |  |  |  |  |
| 8.Aβ phase | **0,028** | 0,960 | **< 0.001** | **< 0.001** | **< 0.001** | **0,001** | **0,040** |  |  |  |  |  |  |  |
| 9.Braak NFT stage | **< 0.001** | 0,721 | **< 0.001** | **< 0.001** | **< 0.001** | **0,004** | **0,016** | **< 0.001** |  |  |  |  |  |  |
| 10.LATE-NC stage | **< 0.001** | 0,623 | **< 0.001** | **< 0.001** | **< 0.001** | **0,014** | 0,365 | **< 0.001** | **< 0.001** |  |  |  |  |  |
| 11. Braak LBD stage | 0,062 | 0,771 | **0,007** | 0,204 | 0,178 | 0,060 | 0,305 | 0,561 | 0,056 | **0,040** |  |  |  |  |
| 12.CERAD score | **0,016** | 0,556 | **< 0.001** | **< 0.001** | **< 0.001** | **0,004** | **0,038** | **< 0.001** | **< 0.001** | **< 0.001** | 0,379 |  |  |  |
| 13.APOEε4 status | **0,021** | 0,402 | **< 0.001** | **< 0.001** | **0,009** | **0,045** | 0,530 | **< 0.001** | **0,001** | **0,020** | **0,047** | **< 0.001** |  |  |
| 14.APOEε2 status | **0,029** | 0,064 | 0,581 | 0,410 | 0,234 | 0,993 | 0,499 | 0,182 | 0,363 | 0,631 | 0,706 | 0,461 | 0,586 |  |
| 15.NIA-AA score | **0,001** | 0,665 | **< 0.001** | **< 0.001** | **< 0.001** | **0,001** | **0,008** | **< 0.001** | **< 0.001** | **< 0.001** | 0,091 | **< 0.001** | **0,001** | 0,347 |

**Supplementary Table 14** – One-way ANOVA with Tuckey’s multiple comparisons test, comparing p-tau seeding in a tau biosensor cell line treated with different brain-derived homogenates (entorhinal cortex, n=34 cases, n=3 biological repeats, n=3 experimental repeats).

| Tukey's multiple comparisons test | Mean Diff, | 95,00% CI of diff, | Adjusted P Value |
| --- | --- | --- | --- |
| Controls vs. AD(LATE-NC-) | -0,2469 | -0,7716 to 0,2779 | 0,5828 |
| Controls vs. AD(LATE-NC+) | -1,934 | -2,417 to -1,451 | **<0,0001** |
| Controls vs. FTLD-TDP | -0,1215 | -0,7274 to 0,4844 | 0,9471 |
| AD (LATE-NC-) vs. AD(LATE-NC+) | -1,687 | -2,201 to -1,173 | **<0,0001** |
| AD(LATE-NC-) vs. FTLD-TDP | 0,1254 | -0,5053 to 0,7560 | 0,9484 |
| AD(LATE-NC+) vs. FTLD-TDP | 1,812 | 1,216 to 2,409 | **<0,0001** |

**Supplementary Table 15** – One-way ANOVA with Tuckey’s multiple comparisons test, comparing p-tau seeding in a tau biosensor cell line treated with different brain-derived homogenates (frontal cortex, n=34 cases, n=3 biological repeats, n=3 experimental repeats).

| Tukey's multiple comparisons test | Mean Diff, | 95,00% CI of diff, | Adjusted P Value |
| --- | --- | --- | --- |
| Controls vs. AD(LATE-NC-) | -0,2469 | -0,7716 to 0,2779 | 0,5828 |
| Controls vs. AD(LATE-NC+) | -1,934 | -2,417 to -1,451 | **<0,0001** |
| Controls vs. FTLD-TDP | -0,1215 | -0,7274 to 0,4844 | 0,9471 |
| AD(LATE-NC-) vs. AD(LATE-NC+) | -1,687 | -2,201 to -1,173 | **<0,0001** |
| AD(LATE-NC-) vs. FTLD-TDP | 0,1254 | -0,5053 to 0,7560 | 0,9484 |
| AD(LATE-NC+) vs. FTLD-TDP | 1,812 | 1,216 to 2,409 | **<0,0001** |

**Supplementary Table 16 –** Multiple linear regression, using the hAP score (ipsilateral) as dependent variable and injection groups as independent variables. Number of animals per injection group: control, n=9, AD(LATE-NC+), n=10 and AD(LATE-NC-), n=9.

| Independent variables | Estimate | Standard error | 95% CI (asymptotic) | \|t\| | P value |
| --- | --- | --- | --- | --- | --- |
| Control | -80,99 | 13,41 | -108,7 to -53,32 | 6,04 | **<0,0001** |
| AD(LATE-NC-) | -10,53 | 13,41 | -38,20 to 17,14 | 0,7856 | 0,4398 |
| Reference group: AD(LATE-NC+) | | | | | |
| Degrees of Freedom: 24 | | | | | |
| R squared: 0,6426 | | | | | |

**Supplementary Table 17 –** Multiple linear regression, using the hAP score (ipsilateral) as dependent variable and injection groups as independent variables. Number of animals per injection group: control, n=9, AD(LATE-NC+), n=10 and AD(LATE-NC-), n=9.

| Independent variables | Estimate | Standard error | 95% CI (asymptotic) | \|t\| | P value |
| --- | --- | --- | --- | --- | --- |
| AD(LATE-NC-) | 70,45 | 13,41 | 42,78 to 98,13 | 5,255 | **<0,0001** |
| AD(LATE-NC+) | 80,99 | 13,41 | 53,32 to 108,7 | 6,04 | **<0,0001** |
| Reference group: Control | | | | | |
| Degrees of Freedom: 24 | | | | | |
| R squared: 0,6426 | | | | | |

**Supplementary Table 18 –** Multiple linear regression, using the hAP score (contralateral) as dependent variable and injection groups as independent variables. Number of animals per injection group: control, n=9, AD(LATE-NC+), n=10 and AD(LATE-NC-), n=9.

| Independent variables | Estimate | Standard error | 95% CI (asymptotic) | \|t\| | P value |
| --- | --- | --- | --- | --- | --- |
| AD(LATE-NC-) | 19,49 | 11,14 | -3,503 to 42,49 | 1,75 | **0,093** |
| AD(LATE-NC+) | 31,04 | 11,14 | 8,046 to 54,04 | 2,786 | **0,0103** |
| Reference group: Control | | | | | |
| Degrees of Freedom: 24 | | | | | |
| R squared: 0,6426 | | | | | |

**Supplementary Table 19 –** Multiple linear regression, using the p-tau202/205 seeding severity (ipsilateral) as dependent variable and injection groups as independent variables. Number of animals per injection group: control, n=9, AD(LATE-NC+), n=10 and AD(LATE-NC-), n=9.

| Independent variables | Estimate | Standard error | 95% CI (asymptotic) | \|t\| | P value |
| --- | --- | --- | --- | --- | --- |
| Control | -2706 | 697,7 | -4147 to -1266 | 3,879 | **0,0007** |
| AD(LATE-NC-) | -1802 | 697,7 | -3243 to -362,4 | 2,583 | **0,0163** |
| Reference group: AD(LATE-NC+) | | | | | |
| Degrees of Freedom: 24 | | | | | |
| R squared: 0,3939 | | | | | |

**Supplementary Table 20 –** Multiple linear regression, using the p-tau202/205 seeding severity (contralateral) as dependent variable and injection groups as independent variables. Number of animals per injection group: control, n=9, AD(LATE-NC+), n=10 and AD(LATE-NC-), n=9.

| Independent variables | Estimate | Standard error | 95% CI (asymptotic) | \|t\| | P value |
| --- | --- | --- | --- | --- | --- |
| Control | -409,8 | 150,8 | -721,1 to -98,49 | 2,717 | **0,012** |
| AD(LATE-NC-) | -332,4 | 150,8 | -643,7 to -21,15 | 2,204 | 0,0373 |
| Reference group: AD(LATE-NC+) | | | | | |
| Degrees of Freedom: 24 | | | | | |
| R squared: 0,2578 | | | | | |

**Supplementary Table 21 –** Multiple linear regression, using TDP-43 nuclear clearance (CA1, ipsilateral) as dependent variable and injection groups as independent variables. Number of animals per injection group: control, n=9, AD(LATE-NC+), n=10 and AD(LATE-NC-), n=9, FTLD-TDP, n=7 and PBS, n=7.

| Independent variables | Estimate | Standard error | 95% CI (asymptotic) | \|t\| | P value |
| --- | --- | --- | --- | --- | --- |
| Control | -12,28 | 5,123 | -22,63 to -1,927 | 2,397 | 0,0213 |
| AD(LATE-NC-) | -13,87 | 4,97 | -23,91 to -3,825 | 2,791 | 0,008 |
| FTLD-TDP | -6,056 | 5,313 | -16,79 to 4,682 | 1,14 | 0,2611 |
| PBS | -11,84 | 5,556 | -23,07 to -0,6089 | 2,131 | 0,0393 |
| Non-injected | -15,44 | 5,313 | -26,18 to -4,703 | 2,906 | 0,0059 |
| Reference group: AD(LATE-NC+) | | | | | |
| Degrees of Freedom: 40 | | | | | |
| R squared: 0,2405 | | | | | |

**Supplementary Table 22 –** Multiple linear regression, using TDP-43 nuclear clearance (motor cortex, ipsilateral) as dependent variable and injection groups as independent variables. Number of animals per injection group: control, n=9, AD(LATE-NC+), n=10 and AD(LATE-NC-), n=9, FTLD-TDP, n=7 and PBS, n=7.

| Independent variables | Estimate | Standard error | 95% CI (asymptotic) | \|t\| | P value |
| --- | --- | --- | --- | --- | --- |
| Control | -20,91 | 8,432 | -37,95 to -3,871 | 2,48 | 0,0174 |
| AD(LATE-NC-) | -26,66 | 8,18 | -43,20 to -10,13 | 3,259 | 0,0023 |
| FTLD-TDP | -18 | 8,745 | -35,68 to -0,3305 | 2,059 | 0,0461 |
| PBS | -27,57 | 9,146 | -46,06 to -9,091 | 3,015 | 0,0044 |
| Non-injected | -22,92 | 8,745 | -40,59 to -5,244 | 2,621 | 0,0123 |
| Reference group: AD(LATE-NC+) | | | | | |
| Degrees of Freedom: 24 | | | | | |
| R squared: 0,6426 | | | | | |

**Supplementary Table 23 –** Multiple linear regression, using TDP-43 nuclear clearance (CA1, ipsilateral) as dependent variable and injection groups as independent variables. Number of animals per injection group: control, n=9, AD(LATE-NC+), n=10 and AD(LATE-NC-), n=9, FTLD-TDP, n=7 and PBS, n=7.

| Independent variables | Estimate | Standard error | 95% CI (asymptotic) | \|t\| | P value |
| --- | --- | --- | --- | --- | --- |
| Control | 3,16 | 5,456 | -7,867 to 14,19 | 0,5792 | 0,5657 |
| AD(LATE-NC-) | 1,571 | 5,313 | -9,167 to 12,31 | 0,2957 | 0,769 |
| AD(LATE-NC+) | 15,44 | 5,313 | 4,703 to 26,18 | 2,906 | 0,0059 |
| FTLD-TDP | 9,385 | 5,635 | -2,004 to 20,77 | 1,665 | 0,1037 |
| PBS | 3,602 | 5,865 | -8,252 to 15,46 | 0,6141 | 0,5426 |
| Reference group: Non-injected | | | | | |
| Degrees of Freedom: 40 | | | | | |
| R squared: 0,2405 | | | | | |

**Supplementary Table 24 –** Multiple linear regression, using neuronal density (CA1, ipsilateral) as dependent variable and injection groups as independent variables. Number of animals per injection group: control, n=9, AD(LATE-NC+), n=10 and AD(LATE-NC-), n=9, FTLD-TDP, n=7 and PBS, n=7.

| Independent variables | Estimate | Standard error | 95% CI (asymptotic) | \|t\| | P value |
| --- | --- | --- | --- | --- | --- |
| Control | 15,41 | 48,97 | -83,48 to 114,3 | 0,3148 | 0,7545 |
| AD(LATE-NC-) | 70,01 | 48,97 | -28,89 to 168,9 | 1,43 | 0,1604 |
| FTLD-TDP | 85,29 | 52,35 | -20,43 to 191,0 | 1,629 | 0,1109 |
| PBS | 7,614 | 54,75 | -103,0 to 118,2 | 0,1391 | 0,8901 |
| Non-injected | 84,1 | 52,35 | -21,63 to 189,8 | 1,606 | 0,1159 |
| Reference group: AD(LATE-NC+) | | | | | |
| Degrees of Freedom: 41 | | | | | |
| R squared: 0,1227 | | | | | |

**Supplementary Table 25 –** Multiple linear regression, using neuronal density (motor cortex, ipsilateral) as dependent variable and injection groups as independent variables. Number of animals per injection group: control, n=9, AD(LATE-NC+), n=10 and AD(LATE-NC-), n=9, FTLD-TDP, n=7 and PBS, n=7.

| Independent variables | Estimate | Standard error | 95% CI (asymptotic) | \|t\| | P value |
| --- | --- | --- | --- | --- | --- |
| Control | -32,68 | 125,6 | -286,3 to 220,9 | 0,2603 | 0,796 |
| AD(LATE-NC-) | 205 | 125,6 | -48,58 to 458,6 | 1,633 | 0,1102 |
| FTLD-TDP | 222,4 | 134,2 | -48,69 to 493,5 | 1,657 | 0,1052 |
| PBS | 54,97 | 140,4 | -228,6 to 338,5 | 0,3915 | 0,6974 |
| Non-injected | 396,8 | 134,2 | 125,7 to 667,9 | 2,956 | 0,0052 |
| Reference group: AD(LATE-NC+) | | | | | |
| Degrees of Freedom: 41 | | | | | |
| R squared: 0,2619 | | | | | |
